# Size control over metal–organic framework porous nanocrystals†
†Electronic supplementary information (ESI) available: Compiled literature size data and experimental conditions. See DOI: 10.1039/c9sc03802g


**DOI:** 10.1039/c9sc03802g

**Published:** 2019-09-12

**Authors:** Checkers R. Marshall, Sara A. Staudhammer, Carl K. Brozek

**Affiliations:** a Department of Chemistry & Biochemistry , Materials Science Institute , University of Oregon , Eugene , Oregon 97403 , USA . Email: cbrozek@uoregon.edu

## Abstract

Porous nanocrystals of metal–organic frameworks (MOFs) offer greater bioavailability, higher surface-to-volume ratios, superior control over MOF membrane fabrication, and enhanced guest-sorption kinetics compared to analogous bulk phases, but reliable synthesis of uniformly sized particles remains an outstanding challenge. Here, we identify the smallest and most probable sizes of known MOF nanocrystals and present an exhaustive comparative summary of nano- *versus* bulk-MOF syntheses. Based on critical analysis of reported size data and experimental conditions, an alternate to the LaMer model is proposed that describes nanocrystal formation as a kinetic competition between acid-base and metal–ligand reactivity. Particle growth terminates when ligands outcompete metal-ion diffusion, thereby arresting polymerization to produce kinetically trapped particle sizes. This model reconciles disparate trends in the literature and postulates that minimum particle sizes can be achieved by minimizing the relative ratios of metal-to-linker local concentrations. By identifying conditions that disfavor small nanocrystal sizes, this model also provides routes towards macroscopic MOF single crystals. A universal “seesaw” relationship between nanocrystal sizes and the concentrations of acidic surface-capping ligands provides a roadmap for achieving precise synthetic control. Best practices in synthesis, characterization, and data presentation are recommended for future investigations so that MOF nanocrystals may achieve their full potential as advanced nanomaterials.

## Abstract 2

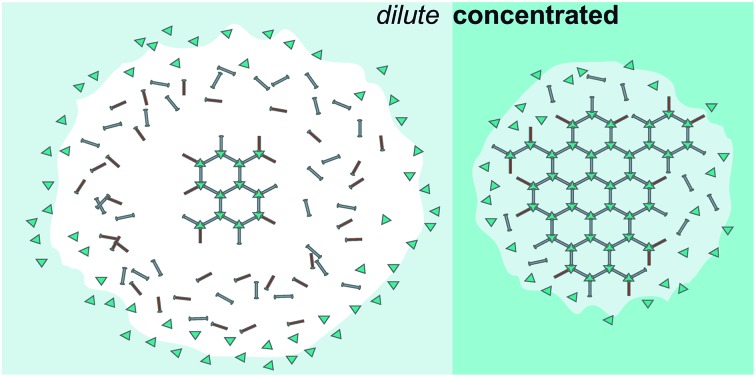
A new model of MOF nanocrystal growth is proposed based on critical analysis of all reported sizes and synthetic conditions.

## Introduction

Nanocrystals are distinguished from their bulk counterparts by the extreme size-dependence of their functional properties. For example, catalytic activities of metal nanoparticles,^1^ nanocrystal plasmon resonance energies,^2^,^3^ and quantum dot absorption and emission profiles in photovoltaic, solar fuel, and luminescence technologies^4^–^6^ reflect underlying electronic structures sensitive to sub-nanometre size variations. Tailoring nanocrystals to a given application therefore relies on generating particles with precise diameter values and uniform size distributions. Since the advent of reliable synthetic methods, inorganic nanocrystals of metals^7^ and semiconductors^8^–^10^ have found widespread use as advanced materials in diverse areas, whereas design principles for organic–inorganic hybrid nanomaterials are just emerging.

Recently, considerable efforts have focused on exploring the nanoscale synthesis of metal–organic frameworks (nano-MOFs) due to the promise of their heightened performance in drug delivery,^11^–^13^ catalysis,^14^ membrane design for gas storage and separation,^15^–^17^ and analyte sensing.^18^ As 3D porous coordination polymers comprised of inorganic clusters bridged by multi-topic organic linkers, MOFs display immense modularity that has given rise to more than 20 000 unique bulk phases,^19^ each with the potential to adopt enhanced functionalities when prepared as nanocrystals.^20^ To advance this research frontier, we must identify synthetic targets and universal mechanistic principles. Building on the publication of recent reviews^21^–^24^ and rigorous mechanistic studies,^25^–^29^ we identified key open questions: *Which MOFs have been prepared as nanocrystals? Which sizes are achievable?* And *Which mechanistic parameters govern nano-MOF sizes?* Here, we address these outstanding questions by compiling experimental parameters and particle sizes from across the nano-MOF literature; statistically treating reported size data (see Methods section below); comparing nano-MOF sizes, size-measurement techniques, and synthetic conditions; and identifying underlying chemical principles from observed trends. Whereas recent reviews^21^,^30^,^31^ have compared the impacts of varying synthetic techniques, such as microwave *versus* solvothermal, and conditions, such as time and temperature, we target the generalized chemical equilibria and kinetic pathways universal to nano-MOF syntheses.

**Fig. 1:**
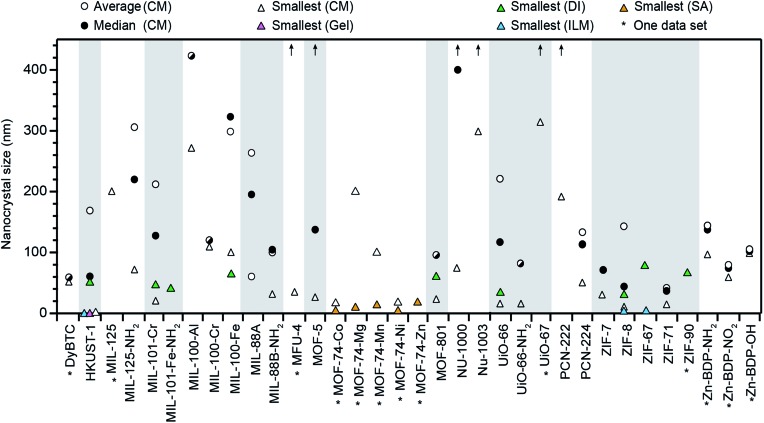
Summary of all MOF materials reported to-date as nanocrystals with precisely measured particle diameters. Average and median sizes are included using all reported literature values for each MOF material. Average sizes for MFU-4, MOF-5, NU-1000, NU-1003, UiO-67, and PCN-222 are above 450 nm, as indicated by arrows. Smallest known sizes for each MOF are labelled according to the corresponding synthetic method, *i.e.*, coordination modulation (CM), metal–organic gel (gel), slow addition (SA), and ionic liquid microemulsions (ILM). See Methods section for details of data treatment. All tabulated values are included in Table S1 of the ESI.†

Fig. 1 summarizes all nano-MOFs we identified with quantifiable size diameters, plotted by average, median, and smallest sizes (listed in Table S1†), and in Table 1 with MOF compositions and experimental details. These data indicate that while many MOF materials have been accessed as nanocrystals, the vast majority have not. Furthermore, Fig. 1 suggests that typical nano-MOF sizes exist on the 100 nm scale, with few extending below 20 nm, in contrast to the 1–10 nm diameters achievable for inorganic nanocrystals.^8^ For most MOF materials, select studies have achieved sub-100 nm diameters, but these cases are exceptions, as size averages and median values are far larger. For each class of MOF materials displayed in Fig. 1, the smallest size provides the current state-of-the-art in minimizing nanocrystal sizes, median values indicate the most likely achievable sizes when using the coordination modulation synthetic method, and average values lend insight into the distribution of reported values for each given class of MOF nanocrystals.

**Table 1:** Common MOF names with chemical formulas, linkers, metal sources, and relevant modulators

MOF Name	MOF molecular formula	Linker	Metal	Effective modulators
COMOC-4, MOF-253	Ga(OH)(BPYDC)	2,2′-Bipyrimidine-5,5′-dicarboxylic acid (BPYDC)	GaNO_3_·H_2_O	None^81^
DUT-23	[Cu_2_(BPY)]_3_(BTB)_4_	4,4,4′′-Benzene-1,3,5-triyl-tribenzoic acid (BTB), 4-4′-Bipyrimidine (BPY)	CuNO_3_·H_2_O	None^82^
DyBTC	Dy(BTC)(H_2_O)·DMF	1,3,5-Benzenetricarboxylic acid	DyNO_3_·H_2_O	Acetic acid, sodium acetate^83^
Fe-soc-MOF	[Fe_3_(μ_3_-O)(H_2_O)_2_(TCPT)1.5Cl]	3,3′,5,5′-Azobenzenetetracarboxylic acid (TCPT)	Fe(NO_3_)_3_·9H_2_O	Sorbitan trioleate (tween-85),^84^ oleic acid + sodium oleate^85^
HKUST-1, Cu-BTC, MOF-199	Cu_3_(BTC)_2_(H_2_O)_3_	1,3,5-Benzenetricarboxylic acid (BTC)	Cu(NO_3_)_2_·3H_2_O, Cu(Oac)_2_·H_2_O	Dodecanoic acid,^44^ sodium acetate, sodium formate, triethylamine,^86^ sodium acetate + triethylamine,^87^ 2-methylimidazole,^88^ PAA^89^
IR-MOF-3	Zn_4_O(TPDC)_3_	2-Aminoterepthalic acid (TPDC)	Zn(NO_3_)_2_·6H_2_O	PVP^89^
				PVP + TMAB^90^
MFU-4	[Zn_5_Cl_4_(BBTA)_3_]·DMF	1*H*,5*H*-Benzo(1,2-*d*:4,5-*d*′)bistriazole (BBTA)	ZnCl_2_·H_2_O	Lutidine^91^
MFU-4l	Zn_5_Cl_4_(BTDD)_3_	Bis(1*H*-1,2,3-triazolo[4,5-*b*],[4′,5′-*i*])dibenzo[1,4]dioxin) (BTDD)	ZnCl_2_·H_2_O	NaOH, KOH^12^
MIL-88A	Fe_3_O(MeOH)_3_(O_2_CCH=CHCO_2_)_3_·MeCO_2_·*n*H_2_O	Fumaric acid	FeCl_3_·6H_2_O	NaOH, acetic acid,^92^ acetic acid, formic acid^93^
MIL-88B-Fe	[Fe_3_O(BDC)_3_(H_2_O)_2_(X)]_*n*_	1,4-Benzenedicarboxylic acid (BDC)	FeCl_3_·6H_2_O	Acetic acid^94^
MIL-88B-NH_2_	[Fe_3_O(BDC-NH_2_)_3_(H_2_O)_2_(X)]_*n*_	1,4-Benzenedicarboxylic acid (BDC)	FeCl_3_·6H_2_O	Acetic acid, F127 55
MIL-96	Al_12_O-(OH)_16_(H_2_O)_5_[BTC]_6_·H_2_O	1,3,5-Benzenetricarboxylic acid (BTC)	Al(NO_3_)_3_·9H_2_O	Trimethyltrimesate,^95^ CTAB, TMAOH^58^
MIL-100-Al	Al_3_·(H_2_O)_2_O(BTC)]_2_·*n*H_2_O	1,3,5-Benzenetricarboxylic acid (BTC)	Al(NO_3_)_3_·9H_2_O	Benzoic acid, trimethyltrimesate^59^
MIL-100-Cr	Cr_3_·(H_2_O)_2_O[(C_6_H_3_)-(CO_2_)_3_]_2_·H_2_O	1,3,5-Benzenetricarboxylic acid (BTC)	Cr(NO_3_)_3_·9H_2_O	None^59^,^96^
MIL-100-Fe	Fe_3_·(H_2_O)_2_O[(C_6_H_3_)-(CO_2_)_3_]_2_·H_2_O	1,3,5-Benzenetricarboxylic acid (BTC)	FeCl_3_·6H_2_O	None^59^,^79^,^97^
MIL-101-Cr	Cr_3_(O)·(BDC)_3_(H_2_O)_2_	1,4-Benzenedicarboxylic acid (BDC)	Cr(NO_3_)_3_·9H_2_O	None, stearic acid, 4-methoxybenzoic acid, benzoic acid, 4-nitrobenzoic acid, perfluorobenzoic acid^98^ acetic acid,^49^ benzoic acid, HF, TMAOH, sodium hydroxide^50^ benzoic acid,^48^
MIL-101-Fe	Fe_3_(O)·(BDC)_3_(H_2_O)_2_	1,4-Benzenedicarboxylic acid (BDC)	FeCl_3_·6H_2_O	2-Methylimidazole^99^
MIL-125	Ti_8_O_8_(OH)_4_(BDC)_6_	1,4-Benzenedicarboxylic acid (BDC)	Ti(OCH(CH_3_)_2_)_4_	Poly(ethylene glycol) diglycidyl ether^100^
MIL-125-NH_2_	Ti_8_O_8_(OH)_4_(BDC-NH_2_)_6_	2-Aminoterephthalic acid (BDC-NH_2_)	Ti(OCH(CH_3_)_2_)_4_	Benzoic acid, thioglycolic acid, acetic acid, *p*-toluic acid^101^
MOF-5, IR-MOF-1	Zn_4_O(BDC)_3_	1,4-Benzenedicarboxylic acid (BDC)	ZnNO_3_·6H_2_O	Acetate (counterion)^83^
				Decylbenzoic acid^102^
				TEA^103^ TEA + PVP^104^ TEA + CTAB^46^ p-perfluoromethylbenzenecarboxylate^26^
MOF-74, CPO-27	M_2_(H_4_DOBDC)	2,5-Dihydroxyterephthalate (DOBDC)	Cu(NO_3_)_2_·3H_2_O, Ni(NO_3_)_2_·6H_2_O	Benzoic acid, acetic acid^105^ TEA,^106^ 2-methylimidazole^99^
MOF-801	Zr_6_O_4_(OH)_4_(C_4_H_2_O_4_)_6_	Fumaric acid	ZrOCl_2_	Acetic acid^107^ benzoic acid^108^ formic acid, trifluoroacetic acid, acetic acid^109^
NU-1000	Zr_6_(μ_3_-OH)_8_(OH)_8_(TBAPy)_2_	(1,3,6,8-Tetrakis(*p*-benzoic acid)pyrene (TBAPy)	ZrOCl_2_	Benzoic acid + trifluoroacetic acid^110^,^111^ biphenyl-4-carboxylic acid^45^ acetic acid^13^
NU-1003	(Zr_6_(μ_3_-OH)_8_(OH)_8_(TNAPy)_2_	1,3,6,8-Tetra(6-carboxynaphthalen-2-yl)pyrene (TNAPy)	ZrOCl_2_	Benzoic acid + trifluoroacetic acid^101^
PCN-222, MOF-545	Zr_6_(μ_3_-O)_8_(OH)_8_(TCPP)_2_	Tetrakis(4-carboxyphenyl)porphyrin (TCPP)	ZrOCl_2_	Benzoic acid,^111^ dichloroacetic acid^13^
PCN-224	Zr_6_O_8_(H_2_O)_8_(TCPP-H_2_)_2_	Tetrakis(4-carboxyphenyl)porphyrin (TCPP)	ZrCl_4_	Benzoic acid^53^,^112^
UiO-66	Zr_6_O_6_(BDC)_6_	1,4-Benzenedicarboxylic acid (BDC)	ZrOCl_2_	Trifluoroacetic acid, dichloroacetic acid, acetic acid, formic acid,^72^ benzoic acid, acetic acid^54^
UiO-67	Zr_6_O_6_(BPDC)_6_	Biphenyl-4,40-dicarboxylic acid (BPDC)	ZrOCl_2_	Benzoic acid, acetic acid^54^
ZIF-7	Zn(Bnim)_2_	Benzimidazole (Bnim)	ZnNO_3_·6H_2_O	Polyethyleneimine^113^
ZIF-71	Zn(Hdcim)_2_	4,5,-Dichloroimidazole (Hdcim)	ZnNO_3_·6H_2_O	*n*-Butylamine
ZIF-8	Zn(Hmim)_2_	2-Methylimidazole (Hmim)	ZnNO_3_·6H_2_O	Excess linker^60^*n*-butylamine^37^
Zn-BDP	Zn(BDP)	1,4-Bis(1*H*-pyrazol-4-yl)benzene (H_2_BDP)	Zn(OAc)_2_·2H_2_O	None^115^

**Fig. 2:**
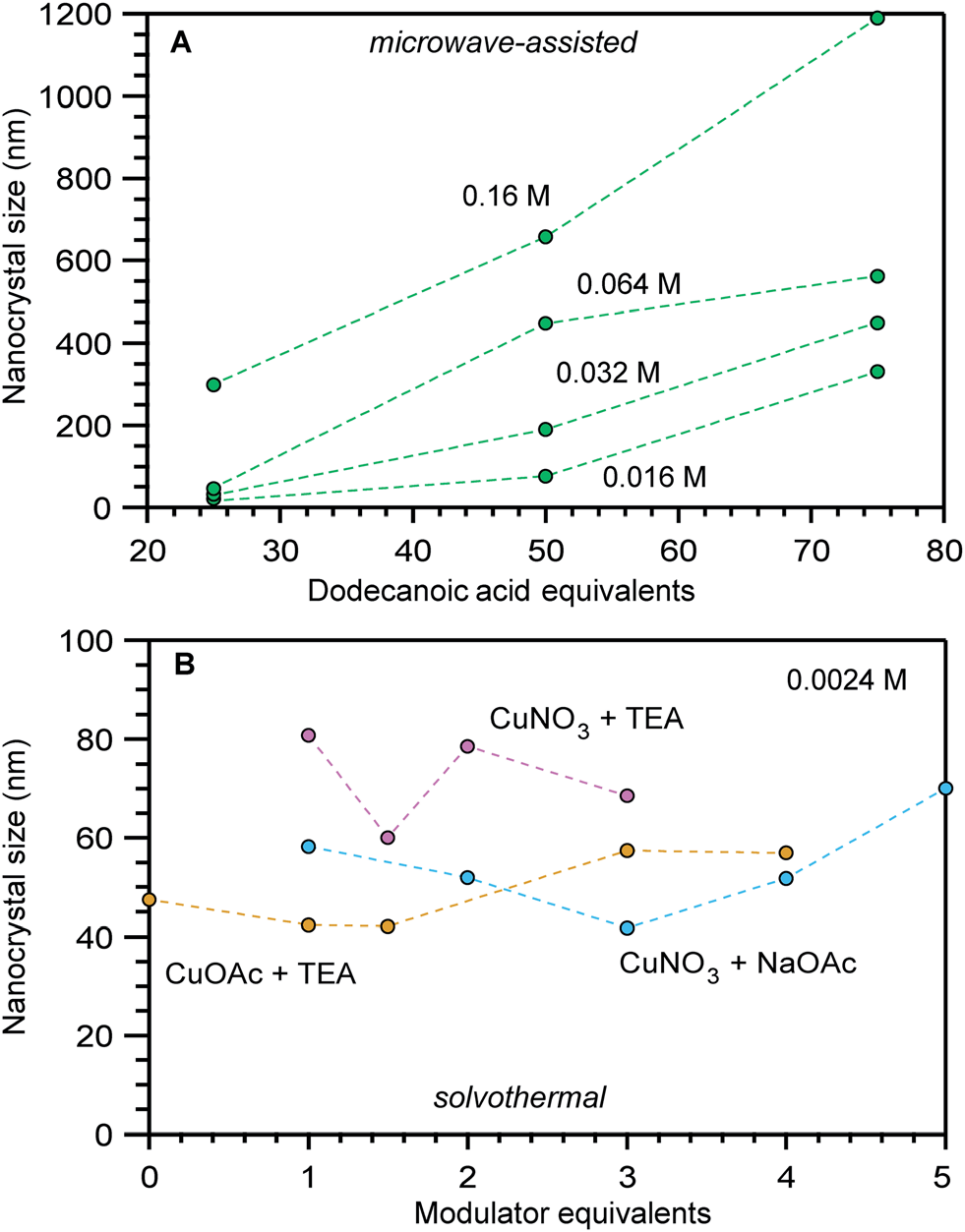
Size comparisons of HKUST-1 nanocrystals prepared by (A) microwave-assisted growth at varying reactant concentrations and added equivalents of dodecanoic acid and (B) by solvothermal synthesis at a fixed reactant concentration of 0.0024 M and varying equivalents of triethylamine (TEA) or acetate (OAc) modulators.^44^,^86^,^87^ The nanocrystal sizes in these studies were determined by TEM (A) and PXRD (B).

Interestingly, compiling size data for a given MOF revealed that often the most impactful size determinants were those that changed between separate synthetic investigations, rather than the parameters systematically explored within isolated studies. For example, Fig. 2 shows a portion of data compiled for nanoscale HKUST-1 (Cu_3_(BTC)_2_(H_2_O)_3_). Clearly, the differing reaction conditions between the results in panel a *versus* panel b had a greater impact on the nanocrystal sizes compared to the minor impact of copper salt and added base identities shown in panel b. Either the differing reactant concentrations (2.34 mM *versus* 0.17 mM), solvent conditions (DMF/H_2_O/EtOH mixture *versus* butanol), or solvothermal *versus* microwave synthetic routes involved distinct processes that produced stark size differences. In response to such cases, we focus our mechanistic analysis on reports that employed “coordination modulators”—typically monotopic acid ligands—as these represent the bulk of literature examples, although small particles of MOFs have been generated by many other techniques, such as preparation *via* microemulsions,^32^,^33^ dual injection,^34^ and metal–organic gels.^35^

Reliable preparation of small nano-MOF particles depends on a firm mechanistic understanding of nano-MOF initiation, growth, and termination. Typically, nano-MOF syntheses are discussed^21^,^23^,^26^ in terms of the LaMer model of particle growth,^36^ which separates crystal nucleation from growth, and describes both in terms of thermodynamic driving forces triggered by high precursor concentrations. *In situ* data suggest that MOF-5 (Zn_4_O(BDC)_3_) may follow this model, as nucleation and growth appear to be effectively separated.^26^ However, systems such as HKUST-1 and ZIF-8 (Zn(Hmim)_2_) behave differently, exhibiting slow nucleation phases that overlap with growth.^37^,^38^ A collection of *in situ* XRD studies of MOF crystal formation revealed no significant difference in the time scales between nucleation and growth phases, implying that both processes can occur simultaneously.^39^ Furthermore, the majority of nano-MOF syntheses occur under dilute conditions (Table S2†). Rather than stabilizing at thermodynamically controlled critical size diameters, termination of nano-MOF growth relies on the presence of capping ligands to surround particle surfaces.

We argue, therefore, that while thermodynamics remain central to understanding MOF crystal nucleation and growth, nano-MOF sizes are kinetically controlled by chemical parameters that arrest particle growth. In particular, *the critical conditions for ensuring small nano-MOF sizes involve depleting the local concentrations of reactant metal ions, thereby allowing linkers and monotopic modulators to trap nano-MOF particles.* Analysis of the literature reveals that ideal conditions involve excess ligand (linker or modulator), dilute reactant concentrations, strong metal–ligand bonds, and low proton activities. In this perspective, we support this kinetic model with literature examples that illustrate the role performed by each parameter and apply this insight to rationalizing previously unexplained phenomena.

## Methods

Most size data shown in the figures, tables, and text of this perspective were reproduced from values enumerated in literature sources, including error bars, which were reported as size deviations. When size ranges were listed without averages, we took the range midpoints as average values (*e.g.*, 100–200 nm would be 150 nm ± 50 nm). When nanoparticle sizes were provided as histograms, average sizes were taken as the modes, with the extreme values as the size distribution ranges. In a few cases, data were digitized from published graphs using the Figure Calibration package in the program ImageJ.^40^ To compile size data, manual searches were conducted in SciFinder, WebofScience and Google Scholar using the terms “nano,” “nanocrystal,” “nanosize,” and “nanoparticle” in addition to the term “MOF” or “metal–organic framework.” To seek specific structures that have been made on the nanoscale, their various common MOF names (*e.g.* CPO-27 or MOF-74) were used in addition to these terms.

For Fig. 1, average and median values were calculated from all compiled literature sources that reported nanocrystals sizes as definitive values (*e.g.*, “∼200 nm” or “about 200 nm” were not used). Sizes reported were analysed regardless of the measurement technique (*i.e.*, PXRD, DLS, *etc*). When multiple size determination techniques were reported for a given nanocrystal investigation, data from those techniques were averaged, then used to determine the global average and median for that MOF material (see Table S3†). The smallest MOF nanocrystals prepared *via* other methods are given in order to compare these values to all MOF nanocrystals obtained by coordination modulation.

### Factors controlling MOF nanocrystal sizes

**Scheme 1:**
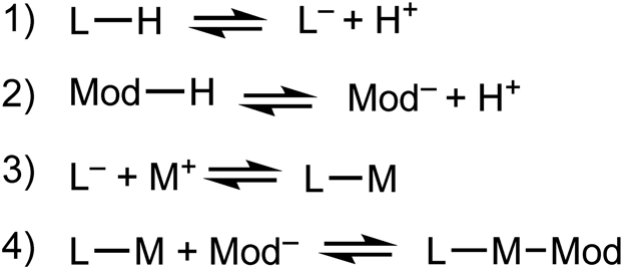
Key chemical equilibria controlling nano-MOF growth and termination.

We propose that the kinetic trapping of MOF nanocrystals of particular sizes depends on the competition between four chemical equilibria (Scheme 1): (1) linker deprotonation; (2) modulator deprotonation; (3) linker complexation, and (4) termination.

Equilibria with fast forward-direction rates and low reversibility dictate whether MOF particles steadily grow toward bulk phases or arrest quickly to form small nanocrystals. MOF linkers must deprotonate (eqn (1)) before forming metal-linker bonds. Modulators are usually acids, and so must also be deprotonated (eqn (2)). Complexation between metal ions and linkers facilitates particle growth (eqn (3)). Reports suggest that early in MOF growth, large collections of molecular complexes and oligomers develop in solution before coalescing into MOF particles.^41^ Subsequent MOF growth is then dominated by the arrival of oligomer clusters or solvated reactant molecules.^42^ During the final termination step (eqn (4)), linker and modulator ligands compete for metal ion coordination sites. According to our kinetic model, this process continues until the local concentration of ligands far exceeds the metal ions, thereby arresting particle growth. In addition to these four chemical processes, the assembly of cluster nodes and solvent decomposition have also been invoked to discuss nano-MOF nucleation and growth,^43^ but we focus on the most general processes that dominate particle trapping. Critical analysis of nano-MOF sizes and synthetic conditions reveal the existence of key parameters that may be programmed to deplete local concentrations of metal ions and generate small particle sizes: modulator identity and concentration, equivalents of linker or modulator, and metal–ligand bond strengths.

#### Modulators

Modulators are typically monotopic carboxylic acids and occasionally Brønsted bases added to nano-MOF syntheses. The intended purpose of modulators varies, but we propose that their function is to influence nano-MOF sizes by affecting linker deprotonation and arresting particle growth.^44^ Modulators also act to prevent particle aggregation. Although modulators produce size trends that appear complex and contradictory, their role can be rationalized in terms of the four equilibria outlined above.

When strong Brønsted bases are used as modulators, their primary role is to facilitate ligand deprotonation (eqn (1)) and enhance metal-linker complexation (eqn (3)) relative to metal-ion diffusion, thereby depleting local metal ion concentrations and forming small MOF nanocrystals. For example, nanocrystals of MFU-4 (Zn_5_Cl_4_(BBTA)_3_) decrease in size with added lutidine or KOH.^12^ Similarly, when nanocrystals of NU-1000 (Zr_6_(μ_3_-OH)_8_(OH)_8_(TBAPy)_2_) are prepared with the addition of 4-biphenyl-carboxylic acid, particle sizes decrease further if NaOH is added to the precursor linker solution^45^ Nanocrystals of MOF-5 and IR-MOF-3 (Zn_4_O(TPDC)_3_) require triethylamine (TEA), which become more uniform with initial addition of cetyltrimethylammonium bromide (CTAB).^46^ Similarly, including *n*-butylamine decreases nanocrystal sizes of ZIF-71 (Zn(Hdcim)_2_).^41^ Interestingly, nanoparticles of MIL-101(Cr) (Cr_3_ (H_2_O)_2_O[(C_6_H_3_)-(CO_2_)_3_]_2_) are synthesized without any modulator by simply decreasing the amount of HF, which is used as a mineralizing agent in the traditional bulk synthesis.^47^–^49^ Adding a strong base to the reaction mixture, however, results in smaller particle sizes.^50^

**Fig. 3:**
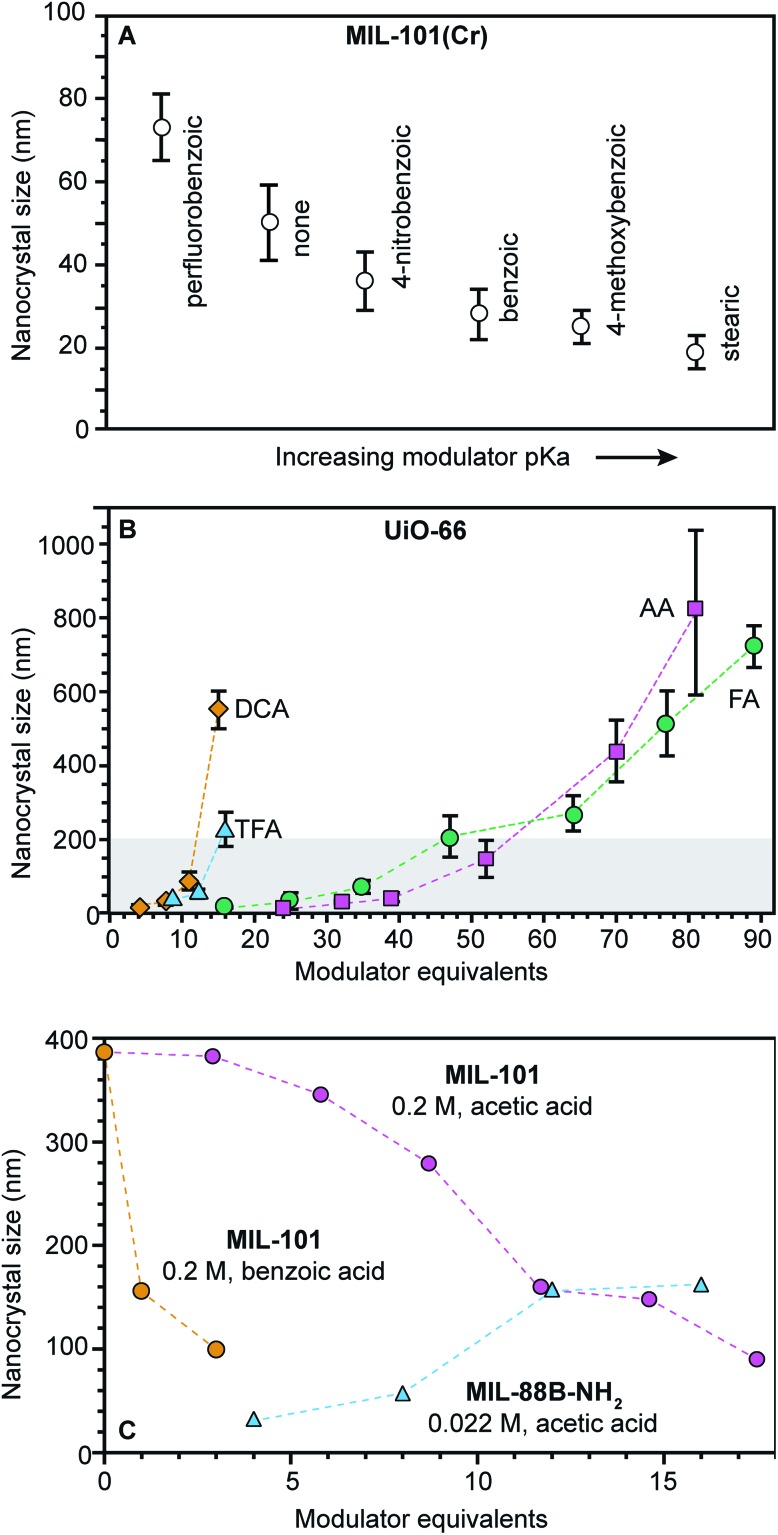
Nanoscale MOF sizes depend on the equivalents and p*K*_a_ values of added modulator reagents. (A) MIL-101(Cr) nanocrystal sizes decrease with increasing modulator p*K*_a_ values. Sizes were determined by TEM.^98^ (B) As modulator equivalents increases, sizes of UiO-66 particles increase. (TFA: trifluoroacetic acid, DCA: dichloroacetic acid, FA: formic acid, and AA: acetic acid). Shaded boxes are provided to emphasize sizes below 200 nm. Sizes were determined with STEM and DLS (DLS not shown).^72^ (C) MIL-101-Cr nanocrystal sizes decrease with increased modulator equivalents, while MIL-88B-NH_2_-Fe exhibits the opposite trend. Interestingly, MIL-88B microcrystals are formed as an impurity at and above 5 benzoic acid equivalents (orange). Sizes were determined with SEM (orange and pink) and TEM (blue).^48^,^49^,^94^

When carboxylic acids serve as modulators, their presence can increase or decrease nano-MOF sizes depending on whether they impede linker deprotonation (eqn (1)) or act as surface capping ligands (eqn (4)). By interfering with deprotonation, they slow down metal-linker complexation (eqn (3)) relative to metal-ion diffusion, resulting in large nano-MOF sizes. On the other hand, they can terminate particle growth by acting as surface-capping ligands and produce small sizes. For example, Fig. 3A shows that while adding 0.33 equivalents of perfluorobenzoic acid generates larger MIL-101 particles relative to using no HF or modulator, the addition of more weakly acidic 4-nitrobenzoic acid, benzoic acid, 4-methoxybenzoic acid, and stearic acid decreases particle sizes with increasing modulator p*K*_a_ values.^51^ The less acidic the modulator, the lower the H^+^ activity in solution available to protonate linker molecules (eqn (1)).

Adding small quantities of acidic modulators decreases nano-MOF sizes until the H^+^ activity in solution reaches a threshold value that begins to interfere with linker deprotonation (eqn (1)). Further addition of acid slows metal–ligand complexation relative to metal-ion diffusion, leading to large particle sizes. For example, Fig. 3A serves as a useful comparison to the data in Fig. 3A. Both studies were conducted at similar concentrations (0.076 M *versus* 0.033 M) and both involve similarly strong metal–ligand bond strengths (Zr^4+^-carboxylate and Cr^3+^-carboxylate) but whereas 0.33 modulator equivalents were employed in Fig. 3A, much higher quantities were involved in Fig. 3B. The data show that UiO-66 (Zr_6_O_6_(BDC)_6_) nanocrystal sizes increase with additional modulator. Interestingly, modulators with lower p*K*_a_ values produce larger particle sizes at a given amount of added modulator. For instance, 15–20 equivalents of trifluoroacetic acid (TFA) or dichloroacetic acid (DCA) produce 200 nm UiO-66 nanocrystal sizes, whereas twice that amount of acetic and formic acid are needed. Acidic modulators slow down metal–ligand complexation (eqn (3)) relative to metal-ion diffusion so that particles continue to grow. Indeed, adding thousands of equivalents of formic acid to the synthesis of UiO-66 generates single crystals hundreds of microns in diameter.^52^ This kinetic explanation fits many other studies in which particle sizes increase with additional acidic modulator,^44^,^53^–^55^ including HKUST-1 modulated by dodecanoic acid,^44^ PCN-224 (Zr-TCPP) with benzoic acid,^53^ UiO-66 with benzoic acid^54^ and MIL-88B-NH_2_ (Fe_3_O(BDC-NH_2_)_3_(H_2_O)_2_) with acetic acid.^55^

Concentrated reaction conditions necessitate the addition of modulator; otherwise, rapid metal-ion diffusion due to short effective pathlengths outcompetes growth termination (eqn (4)). Indeed, most nanoscale MOF syntheses rely on dilute conditions (Table S2†). For example, synthesis of MIL-101-Cr involving high concentrations (0.2 M H_2_BDC) produces small particle sizes only with addition of small quantities of benzoic acid. (Fig. 3C).^48^ The more acidic benzoic acid has a greater effect than acetic acid on decreasing particle sizes at such high reactant concentrations, suggesting that under these reaction conditions, interfering with metal–ligand complexation is critical to kinetically trapping small MIL-101-Cr nanocrystals.

Phase purity must be considered when choosing modulator equivalents and reaction concentrations. For example, while adding few equivalents of either acetic or benzoic acid in the synthesis of MIL-101 at high concentrations results in phase-pure MIL-101 nanocrystals, greater equivalents induce the formation of mixed-phase products^49^ because MIL-101 and MIL-88B occupy the same reaction space, with both arising from Fe^3+^ or Cr^3+^ and trimesic acid.^56^ Therefore, at a benzoic acid : linker ratio of 10 : 1, only MIL-88B microcrystals form.^50^ Concentration plays an important role in controlling nanocrystal phase purity as well. For example, MIL-101-Cr and MIL-88B-Fe nanocrystals have been obtained with similar equivalents of acetic acid, but the synthesis of MIL-88B-Fe was an order of magnitude more dilute (Fig. 3C). Such phase transformations with variable modulator equivalents indicate the importance of nonclassical growth mechanisms.^57^ Similar phenomena have been observed for the phases spaces involving MIL-100-Al (Al_3_·(H_2_O)_2_O(BTC)_2_)/MIL-96-Al (Al_12_O-(OH)_16_(H_2_O)_5_(BTC)_6_ MIL-110-Al (Al_8_(OH)_12_(OH)_3_(H_2_O)_3_(BTC)_3_) and NU-901 (Zr_6_(μ_3_-OH)_8_(OH)_8_(TBAPy)_2_)/NU-1000.^45^,^58^,^59^

#### Linker equivalents

**Fig. 4:**
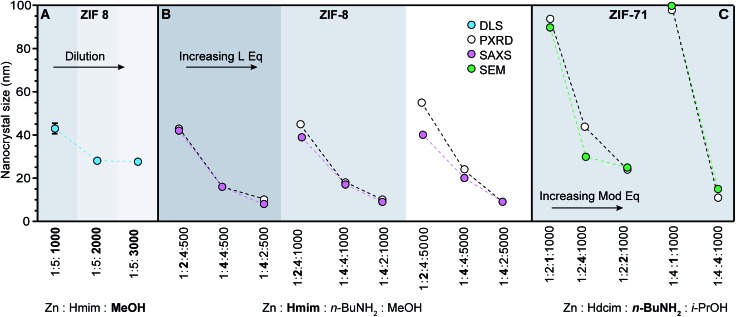
ZIF nanocrystal syntheses with varying relative ratios of metal, linker, modulator, and solvent. Synthetic variables are in bold. (Hmim: 1-methylimidazole, Hdcim: 4,5-dichloroimidazole). (A) Dilution results in a series of ZIF-8 nanocrystals sizes.^60^ (B) Excess linker exerts a stronger influence than base on nanocrystal sizes.^37^ (C) Addition of *n*-butylamine rather than linker excess exhibits biggest impact on ZIF-71 sizes.^41^

Excess linker equivalents shift equilibria toward enhanced metal–ligand complexation (eqn (3)), thereby depleting local metal ion concentrations^44^ and arresting particle growth without added modulator (eqn (4)). In other words, excess linkers serve as surface-capping ligands. The excess linker method was first reported in 2009 for ZIF-8 and has since been used in further ZIF-8 and ZIF-71 nanocrystal syntheses (Fig. 4).^37^,^41^,^60^ Nano-MOF particle sizes can be further reduced by adding Brønsted bases to enhance linker deprotonation (eqn (1)).^60^ Irreversible ligand deprotonation may lead, however, to unchecked particle growth through rapid metal–ligand complexation, unless counterbalanced by excess surface-capping ligands—illustrating the intricate kinetic balance of the four key underlying processes outlined in eqn (1)–(4).

Although several chemical parameters may contribute to decreased nano-MOF sizes, the impact of certain factors may dominate over others. For example, linker excess was discovered to be the single strongest size determinant of ZIF-8 nanocrystals through systematic investigations into the role of Brønsted base, linker excess, and reactant concentrations (Fig. 4A and B).^60^ Nanocrystals of ZIF-8 can be synthesized using an excess of the linker 1-methylimidazolium (Hmim),^60^ whereas typical bulk syntheses of ZIF-8 combine the zinc salt and imidazole linker in a 1 : 1 ratio.^61^ Simply increasing the metal-to-linker ratio to 1 : 5 results in nanocrystals sizes of 40 nm (Fig. 4A).^60^ Reactant concentration was also studied as a size determinant, with the data in Fig. 4A showing that more dilute systems lead to smaller ZIF-8 crystal sizes. In terms of our kinetic model, the role of dilution is to increase metal-ion diffusion pathlengths, allowing particles to be terminated in isolation from additional metal ions. The impact of added base was also investigated, but only the basic modulator *n*-butylamine resulted in reduced nanocrystal sizes, whereas less basic 1-methylimidazole and sodium formate resulted in micrometre-sized crystals.^37^ Nevertheless, compared to the impact of dilution (Fig. 4A) and Brønsted base, the most significant decreases in ZIF-8 sizes were achieved by linker excess (Fig. 4B). These systematic comparisons suggest that growth termination is more important than linker deprotonation in controlling ZIF-8 nanocrystal sizes.

If linker deprotonation limits nanocrystal formation kinetics, however, addition of Brønsted base will produce a greater effect than the equivalents of excess linker. For example, systematic studies of ZIF-71 nanocrystal synthesis indicate that in contrast to ZIF-8, the most influential variable is *n*-butylamine equivalents (Fig. 4C).^41^ When the linker-to-metal ratio is doubled from two to four with base and concentration held constant, particle sizes remain around 80–100 nm. Increasing the proportion of base, however, reduces particle sizes to approximately 20 nm. The sensitivity of ZIF-71 nanocrystal sizes to the equivalents of added base results from the less acidic 4,5-dichloroimidazole linker.

Interestingly, rather than follow this excess linker strategy, most reported nano-MOF syntheses rely on the same linker equivalents used in bulk syntheses (Table S2†). On the other hand, select studies have shown that excess linker was ineffective in generating nanoscale particles. Excess trimesic acid does not produce HKUST-1 nanocrystals, for instance.^38^ Although excess linker reduces the sizes of UiO-66 particles, higher water content exerted the greatest size control, perhaps due to its role in assembling the Zr^4+^-oxo cluster nodes.^62^

#### Metal–ligand bond strengths

**Fig. 5:**
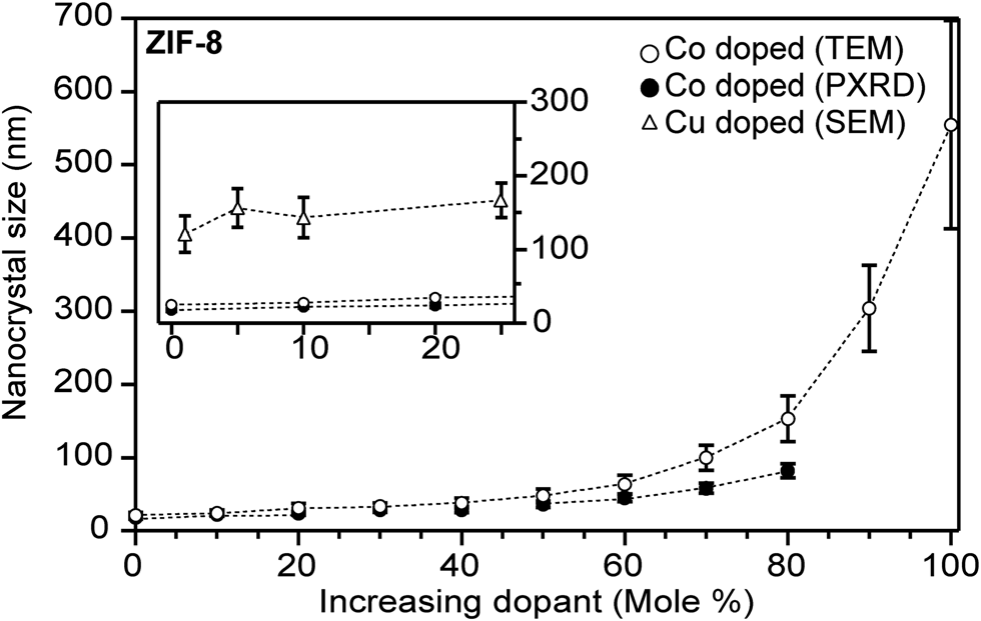
Heterobimetallic ZIF-8 nanocrystals increase in size as the Zn^2+^ atoms are substituted for Co^2+^ or Cu^2+^ atoms. Insert: highlighted data at low equivalents, where identical Co^2+^ and Cu^2+^ quantities produce different particle sizes. Particle sizes were determined by TEM (main) and SEM (insert).^63^,^114^

Strong metal–ligand interactions favour small particle size because they enhance rates of both complexation (eqn (3)) and termination (eqn (4)) during nano-MOF growth, thereby depleting the local concentrations of metal ions relative to linkers or modulators. Systematic studies varying the metal identities of heterobimetallic materials illustrates the influence of metal–ligand interactions on nanocrystal size. For example, higher Co^2+^ contents in Zn^2+^-based ZIF-8 nanocrystals results in larger nanocrystals (Fig. 5).^63^ Using Cu^2+^ further accentuates this effect, with comparatively larger sizes produced at identical dopant metal concentrations.^64^ Because linker-to-metal ratios remained constant in these experiments, the increase in size with lower Zn^2+^ content can be attributed to the strong Zn^2+^-imidazolate interactions, which quickly produce small particles unless harder ions such as Cu^2+^ interfere. Similarly, differences in metal ion labilities were invoked to explain why MOF-74 (M_2_(DOBDC)) crystals nucleate and grow faster with Zn^2+^ than with Co^2+^.^42^ Surprisingly, cobalt-doped UiO-66 nanoparticles are smaller in size than their zirconium-only counterparts when synthesized under otherwise identical conditions.^65^ As the strength of the zirconium–carboxylate bond is expected to be stronger than cobalt-carboxylate bonds, metal-linker complexation rates may not be the only equilibrium to consider. For instance, weaker bonds might slow particle growth, allowing diffusing linkers trap the cobalt variants at smaller sizes. To date, there have been few studies regarding the effect of mixed metals on MOF nanocrystal size and this area warrants further exploration.

#### Summary

**Scheme 2:**
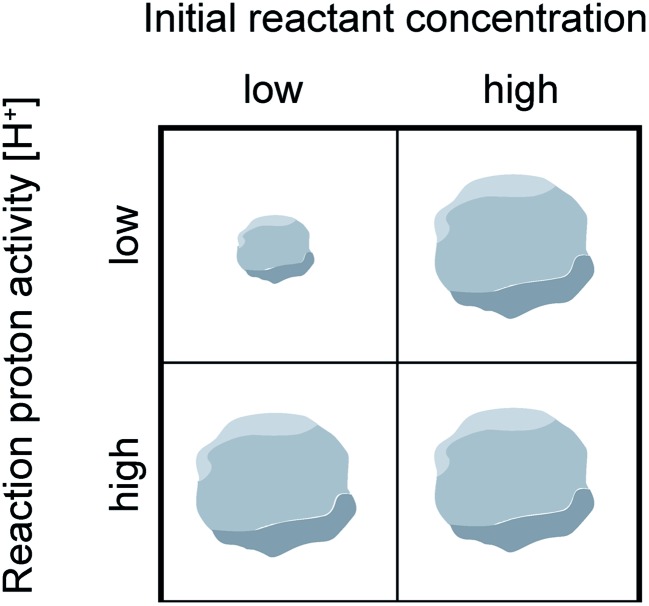
Reaction conditions that favor small or large MOF nanocrystal sizes when linker or acidic modulators are present in excess.

The metal–ligand chemistry outlined in eqn (1)–(4) provides a framework for understanding trends in reported nano-MOF sizes. Based on these insights, Scheme 2 offers a general guide for designing small MOF nanocrystals. Excess linker or acidic modulator generally reduce nanocrystal sizes unless either metal-linker complexation far exceeds termination kinetics or if acid addition inhibits linker deprotonation. Dilute reactant concentrations paired with low proton activities ensure small particle sizes by enhancing complexation (eqn (3)) and termination (eqn (4)), while isolating particles from diffusing metal ions to prevent runaway growth.

## The “Seesaw” relationship of nano-MOF sizes

**Fig. 6:**
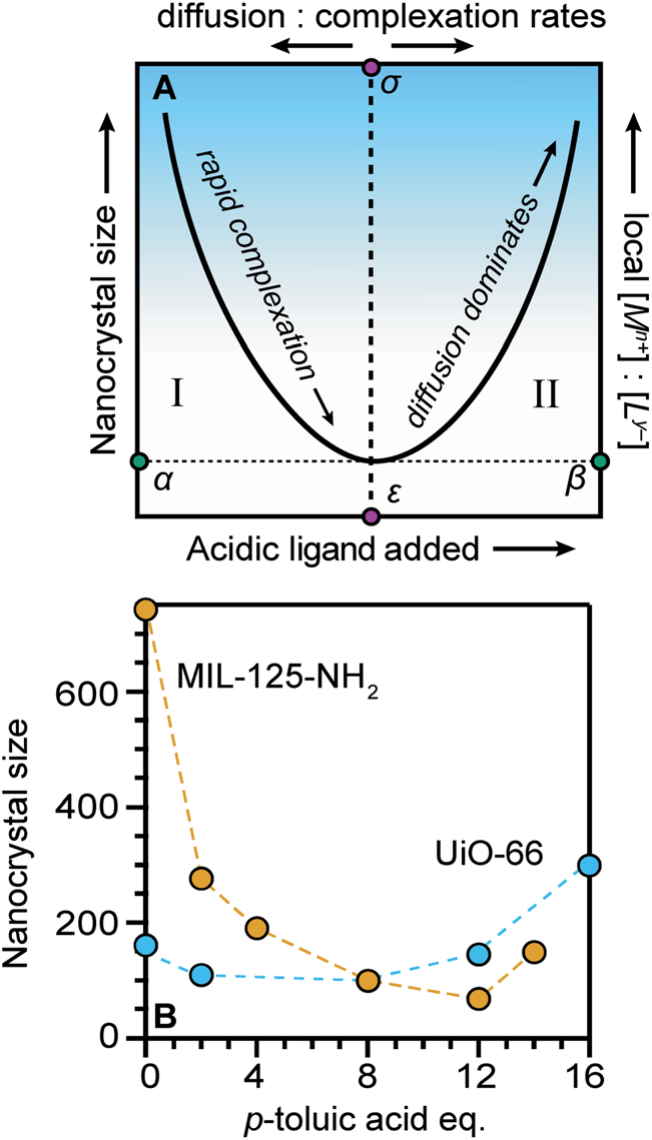
The “Seesaw” relationship between nanocrystal sizes and added equivalents of acidic ligands. Nanocrystal sizes increase with higher ratios of metal-to-linker local concentrations (A) particles reach a minimum size *α* at critical values of acidic ligand *ε* and minimum relative ratios local metal ion-to-ligand concentrations *β* and relative ratios diffusion and metal–ligand complexation ratios *σ*. (B) MIL-125-NH_2_ and UiO-66 exhibit the full seesaw relationship curve in trends between particle sizes and equivalents of *p*-toluic acid.^66^

Seemingly incompatible trends reported for nano-MOF sizes can be reconciled by viewing nano-MOF growth as a balance between reactant concentration, linker and modulator deprotonation, metal–ligand interactions, and metal-ion diffusion. Fig. 6A summarizes our model into two regimes. In regime I, small quantities of acidic ligands (either modulators or linkers) decrease particle sizes by supplying surface-capping ligands, overwhelming local metal ion concentrations. Higher quantities of acidic ligands further decrease nanocrystal sizes by increasing the rate of metal–ligand complexation relative to metal-ion diffusion. This trend continues until reaching minimum nanocrystal sizes *α* at threshold values of added acidic ligand *ε* (Fig. 6A). This critical point corresponds to a minimum of relative ratios between local metal ion-to-ligand concentrations *β* and ratios of relative rates of diffusion and metal–ligand complexation *σ* (Fig. 6A). In regime II, additional equivalents of acidic ligands raise solution proton activities such that they interfere with linker deprotonation. As a result, nanocrystal sizes increase with additional acidic ligand because metal-ion diffusion rates outcompete particle termination. This “seesaw” relationship between nano-MOF sizes and relative termination *versus* diffusion rates strikes a balance precisely where particles sizes are at a minimum.

Fig. 6B summarizes data exhibiting a seesaw curve for NH_2_-MIL-125 and UiO-66 sizes with varying quantities of *p*-toluic acid.^66^ For both materials, particle sizes at first decrease, bottom-out at minimum values, and then increase with higher quantities of modulator. Competition between complexation and metal-ion diffusion may explain seesaw curves in related phenomena, such as the polymorphic balance between MIL-101 and MIL-88B phases achieved by tuning benzoic acid equivalents (Fig. 3C).^48^ Low benzoic acid equivalents produce decreasing sizes of MIL-101 until higher equivalents lead to large micron-sized particles of MIL-88B instead.

We propose that most reported trends of nano-MOF sizes capture just portions of the entire curve of the seesaw relationship. Regime-I behavior, where added acidic ligand decreases particle sizes, is observed for the MIL-101(Cr) and ZIF-8 syntheses discussed above (Fig. 3A and 4A).^48^,^49^,^60^ Examples of regime II behavior, where particle size increases with respect to increasing modulator equivalents, have been observed for many MOFs when monocarboxylic acids are added, including reports on UiO-66, HKUST-1, PCN-224, and MIL-88B-NH_2_.^44^,^53^–^55^ Based on numerous reports exhibiting regime II behavior, the curvature of the slope region II appears proportional to modulator acidity such that highly acidic ligands produce larger particle sizes at fixed equivalents. The impact of highly acidic modulators is so pronounced that they can halt particle growth entirely, whereas less acidic modulators added in large excess simply promote large particles.

Whether regime-I or regime-II behaviors emerge for a given MOF material depends on the similarities between the particular complexation, termination, and metal-ion diffusion rates.

Although the seesaw curve involves kinetic trapping, the extreme limit at the far right of the curve involves particles grow over much longer time periods due to sluggish metal–ligand complexation that places MOF crystal growth in an entirely different regime determined by thermodynamics. According to this model, defects incorporated in nano-MOFs must be kinetically trapped, whereas defects in macroscopic MOF single crystals arrive through thermodynamically driven processes. This model helps explains why addition of strong acid helps to produce large single crystals of MOFs.^67^

## Best practices and outstanding challenges

Elevating the rigor of MOF nanocrystal synthesis will require addressing critical challenges. Here, we offer recommendations on synthetic, characterization, and data-reporting methods to facilitate future MOF nanocrystal investigations.

### Colloidal stability

Applying MOF nanoparticles in applications such as drug delivery requires that the particles be colloidally stable. A re-dispersed nanoparticle solution may suffer from significant aggregation or coalescence without sufficient surface capping ligand coverage. Measurements of zeta potentials provide useful information on the charge at the nanoparticle surface, such that values far away from zero indicate that a dispersion is stable.^68^ DLS (dynamic light scattering) measurements may be used to determine colloidal stability, as it is a solution-phase size measurement method. Aggregating particles observed by DLS display unusually high hydrodynamic radii. Additionally, further growth or aggregation causes the apparent sizes to increase over time.

### Incorporation of modulator

Identifying the presence and location of modulators in nano-MOFs is important in determining whether they serve as surface-capping ligands or form internal defects. Mirkin *et al.* found that while the colloidal stability of UiO-66 crystals correlated to the identity and amount of modulator, the exact role of modulators at the particles surfaces was unclear.^68^ For example, while small equivalents of weakly acidic modulators resulted in aggregation,^68^ nanoparticles of UiO-66 have been synthesized without any monocarboxylic acid modulator.^62^

A common method to quantify ligand incorporation in MOFs is to perform acid digestion NMR studies. The linker-to-modulator ratio in the MOF can be elucidated through ^1^H-NMR peak integration.^69^ Defects may also be identified as a weight percent by thermal gravimetric analysis (TGA).^46^,^70^ The relative incorporation of a modulator depends on its function during synthesis. For instance, in the synthesis of ZIF-8 with *n*-butylamine, less than 1% incorporation is observed by ^1^H NMR. The absence of incorporated modulator indicates its primary role is to deprotonate the linker, rather than cap particles during termination.^71^ When the ratio of modulator is higher than would be expected for surface passivation, it must either be creating defects in the MOF particle, or be present as a guest. For example, a reported synthesis of UiO-66 modulated with benzoic acid revealed an 8 : 10 benzoic acid-to-linker ratio, even after extensive washing.^54^ The amount of modulator incorporated can depend on pH, as one ^1^H NMR digestion study of UiO-66 showed that acetic acid incorporation first decreased, then increased, with respect to the amount of triethylamine added.^69^ The authors speculated that amount of deprotonated BDC in the reaction was maximized at the minimum of the acetate incorporation curve.^69^ Interestingly, modulator incorporation observed in this study exhibits a U-shaped curve, indicating that modulator defect concentrations can be minimized at a critical amount of added modulator. This U-shape does not correspond to size, however; the size monotonically decreases, indicating the minimum size and defect concentration occur with different quantities of added modulator. Due to the insight obtained from these studies, we recommend acid digestion NMR studies as a standard method to characterize MOF nanoparticles. We expect synthetic methods to advance toward finer levels of control as trends emerge from the impact modulators have on defect incorporation and nanocrystal size.

### Measurement methods

Size analysis of MOF nanocrystals relies on appropriate use of structural characterization methods, as has been discussed in a previous review.^27^ Typical techniques include PXRD (powder X-ray diffraction), microscopy, DLS, and SAXS (small angle X-ray scattering). In general, we recommend reporting data from at least two complimentary methods, even when data contradict.

According to the Scherrer relation,^72^ the full-width-at-half-max of a given PXRD peak relates to the particle size. Although smaller particles will exhibit broader diffraction peaks in general, peak broadening may result from several factors, such as lattice stress or instrument effects.^73^ Several peaks should be modelled to determine reliable size estimates. When considering polyhedral crystals, shape factors should be chosen to match the particle morphology and specific miller index of the peak under consideration.^73^ Crystallographic domain, not particle, sizes are estimated by this method. Aggregated particles comprise of multiple domains, which leads to conflicting data between PXRD and other sizing techniques.^74^

Dynamic light scattering (DLS) overestimates particle sizes because the method determines the hydrodynamic radii of particles. Authors often attribute size overestimates from DLS to aggregation. Recent reports have suggested that MOF porosity may induce unconventional diffusion behaviour, which would hamper analysis by DLS.^62^ The interpretation of DLS relies on the assumption that particles are hard spheres that move in solution *via* Brownian motion.^75^ Irregularly shaped particles or porous particles defy these simplified models.^76^,^77^ Several advanced models exist that describe hollow nanoparticles, although these too may be inadequate for describing the complex microporosity of MOF particles.^78^ The key utility of DLS is in developing biological applications of large particles, where it can effectively identify the presence of microscopic aggregates in solution.^79^

Microscopy is the most common method to determine particle sizes. Both SEM (scanning electron microscopy) and TEM (transmission electron microscopy) are widely used, although they rely on high-energy electron beams that can compromise MOF structural integrity.^27^ Microscopy finds its greatest advantage in probing particle morphology, although the 2D projections of 3D particle shapes should be considered carefully.^60^ Furthermore, analysis must be applied to statistically relevant ensembles of particles. It is essential to report the size of the population used to estimate size and size distributions; these values are often missing in the literature.

SAXS is a less common technique, but it presents several advantages: SAXS measures solution-state samples without overestimating sizes and it examines statistically relevant populations.^37^ Accurate analysis relies on choosing appropriate approximations and form factors.^80^ Although size and porosity of hollow nanoparticles can be accurately determined by SAXS, nano-MOFs lack a generally accepted model due to their complex topology.^78^ The model used, and any other relevant data analysis, should be rigorously reported. In general, critical treatment of particle size data is essential to rigorous investigations into the structure–property relationships of MOF nanocrystals.

## Conclusions

MOF nanocrystal sizes and synthetic conditions were critically analysed from across the literature to develop a deeper mechanistic understanding of nanocrystal formation. A general model was presented that reconciles seemingly contradictory trends for MOF nanocrystal sizes *versus* common synthetic parameters: excess ligand, additional acid or base, reactant concentrations, and metal ion identities. A universal “seesaw” relationship is proposed that relates nano-MOF sizes to a competition between particle growth facilitated by diffusing metal ions and particle termination by depleting metal ion local concentrations through rapid ligand complexation. Therefore, conditions that favour high relative concentrations of ligands and that maximize metal-ion diffusion pathlengths produce the smallest nano-MOF sizes. This model also sheds light on the mechanism of MOF crystal growth, in general, and provides a framework for designing macroscopic single crystals. By compiling data for all known MOF nanocrystals, we define the goalposts for future nano-MOF synthetic targets and provide a mechanistic model rooted in chemical parameters that may be tuned to discover the full potential of this emerging class of nanomaterials.

## Conflicts of interest

There are no conflicts to declare.

## Supplementary Material

Supplementary information
