# Supplementary material for: Size control over metal–organic framework porous nanocrystals
Source: Chem Sci. 2019 Sep 12;10(41):9396–408. doi: 10.1039/c9sc03802g (PMC6979335; doi:10.1039/c9sc03802g)
Supplement: Supplementary file 1 [file SC-010-C9SC03802G-s001.pdf]

Electronic Supplementary Material (ESI) for Chemical Science.
This journal is © The Royal Society of Chemistry 2019

Supporting Information

Size Control over Metal-Organic Framework Porous Nanocrystals

Checkers R. Marshall, Sara A. Staudhammer, Carl K. Brozek*
University of Oregon, Eugene

**Email: cbrozek@uoregon.edu*

Contents

Table S1 **2**

Table S2 **3**

Table S3 **6**

References **16**

Table S1: Values for the smallest, median, and average nanocrystal sizes reported in Figure 1 of the main text. The smallest MOF nanocrystals made by other methods are reported along with the method used: metal organic gel (Gel,) ionic liquid microemulsion (ILM), dual injection (Inject), and slow addition (SA)

MOF	Smallest	Median	Average	Smallest by Other Methods	Smallest Ref
DyBTC	50	60	59.2		[1]
HKUST-1	2.6	60	164.71	2.6 – Gel<br>1.6 – ILM<br>24 – Inject	[2–5]
IR-MOF-3	30				[6]
MFU-4	33.66	1200	750.2		[7]
MOF-74	17 (Co)			16.6 (Zn) (SA), 13.6 (Mn) (SA),<br>9.4 (Mg), 5.1 (Co) (SA), 2.8 (Ni) (SA)	[8, 9]
MOF-801	23	95.5	95.5		[10]
MIL-88-A	60 195	263.64			[11]
MIL-88B-NH_2	30	105	100		[12]
MIL-96	930	2100	2795.7		[13]
MIL-100-Al	272	423.5	423.5		[14]
MIL-100-Cr	109.33	119.79	119.79		[15]
MIL-100-Fe	100	323	298.81		[16]
MIL-101-Cr	19	127.5	211.8		[17]
MIL-101-Fe				47 – Inject	[5]
MIL-125	85	550	583.8		[18]
MIL-125-NH_2	70	220	305.6		[19]
MOF-5	25	137.5	709.8		
NU-1000	75	400	1965.5		[20]
NU-1003	300				[21]
PCN-222	190	550	535.8		[22]
PCN-224	49	1141	133.1		[23]
UiO-66	14	117	221.2	35 – Inject	[5, 24]
UiO-66-NH_2	16	82	82		[24]
UiO-67	308	515.5	481		[24]
ZIF-7	30.7	71.4	71.4		[25]
ZIF-8	9	42.3	141.3	32 – Inject<br>2.2 - ILM	[4, 5, 26]
ZIF-67				80 - Inject<br>2.3 - ILM	[4, 5]
ZIF-71	13	37	41.6		[27]
ZIF-90				65 – Inject	[5]
Zn-BPD-H	75.5	100	105.1		[28]
Zn-BPD-NH_2	95	137.5	144.4		[28]
Zn-BPD-NO_2	57.5	79.5	74.6		[28]
Zn-BPD-OH	97	102.5	105.1		[28]

Table S2: Typical M:L ratios and L concentrations for MOF nanocrystals discussed in this perspective. These values are compared to 1-2 representative examples for bulk syntheses. Bold values indicate nanoscale syntheses where either excess linker or a more dilute system was used compared to bulk syntheses.

MOF Name	Bulk L : 1M	Bulk L Conc. (M)	Nano L : 1M	Nano L Conc. (M)	Nano Ref	Bulk Ref
$[(2\text{-PTZ})_2Cd(H_2O)_2]$	0.125 – 6, 0.5 – 4 (M = Zn)	0.66 – 1 (M = Zn)	2, 3	0.2, 0.3	[29]	[30]
COMOC-4	Not available		1.14	0.05	[31]	
DUT-23 (Cu)	0.4	0.0076	**2, 4**	**0.00052, 0.0010**	[32]	[33]
Dy-BTC	1	0.01	**1.5**	0.0125	[1]	[34]
Fe-soc-MOF	2		0.49	0.013	[35, 36]	
HKUST-1	0.67	0.020	0.53	0.04	[1]	[37]
	0.53	0.012	0.67	0.042	[38]	[39]
			0.67	0.04	[40]	
			0.56	variable	[41]	
			0.56	0.099	[42]	
			**1**	0.041	[43]	
IR-MOF-3	0.34	0.04	0.34	**0.043**	[6]	[44]
			0.37	**0.02**	[43]	
MFU-4	0.25	0.0625	0.24	**0.03**	[7]	[45]
MFU-4l	0.048	0.0036	0.05	**0.0019**	[46]	[47]
MIL-100 (Al)	0.67	0.11	0.66	0.241	[15]	[48]
MIL-96 (Al)	0.81	0.14	0.081	**0.01**	[13]	[49]
MIL-100 (Cr)	0.67 (M = Cr(0))	0.14	0.67	0.1333	[15]	[50]
MIL-100 (Fe)	0.66 (M = Fe(0))	0.13	0.67	0.1333	[15]	[51]
			0.67	0.201	[52]	
MIL-101 (Cr)	1	0.21	1	0.2	[53]	[54]
			1	0.2	[55]	
			1	0.033	[17]	
			1	0.2	[56]	
MIL-101-NH_2 (Fe)	0.75	0.017	0.51	0.083	[40]	[6]
MIL-88A	1	0.2	1	0.2	[11]	[57]
MIL-88B (Cr)	1	0.08	1	0.2	[58]	[59]
MIL-88B (Fe)	1	0.046	0.65	0.1	[60]	[61]
MIL-88B-NH2 (Fe)	1	0.046	0.5	**0.022**	[12]	[61]
MIL-125	0.67	0.3	**1.3**	Not available	[62]	
			0.55	**1.44 × 10-7**	[18]	[63]
MIL-125-NH_2	2	0.12	1.5	0.277	[19]	[63]
			1.55	**0.0775**	[64]	
MIL-53 (Al)	0.5	0.347	0.5	0.347	[65]	[66]
MOF-5	0.48	0.024	**0.4 – 1**	0.053 – 0.13	[1]	[67]
	0.337	0.0367	0.2	**0.0067**	[40]	[68]

Table S2: Typical M:L ratios and L concentrations for MOF nanocrystals discussed in this perspective. These values are compared to 1-2 representative examples for bulk syntheses. Bold values indicate nanoscale syntheses where either excess linker or a more dilute system was used compared to bulk syntheses.

MOF Name	Bulk L : 1M	Bulk L Conc. (M)	Nano L : 1M	Nano L Conc. (M)	Nano Ref	Bulk Ref
			0.33	**0.01**	[69]	
			0.5	**0.0005**	[32]	
			1	**0.00166**	[70]	
			3	**0.005**		
			0.43	**0.000125 - 0.0028**	[71]	
			0.33	0.04	[72]	
			0.5	0.05	[73]	
			0.33	0.04	[74]	
MOF-74 (Co)	0.25	0.01	0.28	Not available	[40]	[40]
MOF-74 (Ni)	Not available		0.29	0.012	[40]	
			0.5	0.0417	[75]	
MOF-801	Not available		2	0.18	[10]	
NU-1000	0.2	0.0074	0.088	**0.0014**	[22]	[76]
			0.061	**0.0056**	[77]	
			0.1	**0.0018**	[20]	
			0.0054	**0.0036**	[78]	
PCN-222	0.12	0.0038	0.068	**0.00053**	[22]	[79]
			0.1	**0.0018**	[20]	
PCN-224	0.14	0.0065	0.14	**0.0013**	[23]	
			0.17	**0.002**	[80]	
UiO-66			1	0.0257	[81]	[82]
	1		**1.5**	0.0386		
			**2**	0.0515		
		0.0086	**3.34**	0.075	[83]	
			1	**0.0043**	[84]	
			1	varies	[85]	
			1	0.0172	[24]	
			1	0.0454	[86]	
			1	0.0454	[64]	
			0.74	0.02	[78]	
UiO-66 w/ Co			1	0.078	[87]	
UiO-66-NH_2	1 (X = NH_2)	0.0356				
UiO-67	Not available		0.74	0.02	[78]	
UMCM-150	0.49	0.0070	0.33	**0.00051**	[32]	[88]
ZIF 8 w/ Co	Not available		0.34	0.1	[89]	
			**16**	**0.000099**	[90]	
ZIF-65-Zn	0.5	0.067	2	0.1	[91]	[92]
ZIF-7	0.81	0.027	2	0.2	[91]	[93]
			2	**0.015**	20	
ZIF-71	4	0.053	2	0.2	[91]	[94]

Table S2: Typical M:L ratios and L concentrations for MOF nanocrystals discussed in this perspective. These values are compared to 1-2 representative examples for bulk syntheses. Bold values indicate nanoscale syntheses where either excess linker or a more dilute system was used compared to bulk syntheses.

MOF Name	Bulk L : 1M	Bulk L Conc. (M)	Nano L : 1M	Nano L Conc. (M)	Nano Ref	Bulk Ref
ZIF-8	0.91	0.041	1.9-2	0.05	[89]	
			**8**	0.20, 0.089	[95]	[96]
Zn-BPD-X (X = NH_2, NO_2, OH)	1	0.05	1	0.013	[28]	[97]
Zn-BPD-OH			1	0.027	[28]	

Table S3: Nanocrystal sizes used to create Fig 1 in the main text. Size distributions given in nanometers are shown in parentheses. Dispersity measurements reported as PDI (Polydispersity Index), standard deviation, relative standard deviation, or a range of nanometers are reported in Disp. (Other) with the type of data in parentheses (PDI, SD, RSD, or Range). In cases where anisotropic particles were presented with both length and width, they are differentiated here as (L) and (W).

MOF	Size (nm)	Disp. (Other)	Method	Size	Method	Size	Method	Method Average	Citation
$[(2\text{-PTZ})_2Cd(H_2O)_2]$	Not available								[29]
BUT-12	Not available					[98]			
COMOC-4 (w/ Eu)	Not available								[31]
DUT-23 Cu	Not available								[32]
Dy-BTC	70(15)		SEM						
	50(30)		SEM						
	50(25)		SEM						
	65(25)		SEM						
	60(20)		SEM						
	60(20)		SEM						
Fe-soc-MOF	310(10)		SEM						
HKUST-1	100		DLS						[43]
	47		DLS						
	48		DLS						
	60		DLS						
	54		DLS						
	20000		SEM						[99]
	300		SEM						
	85		SEM						
	100		SEM						
	115		SEM						
	600		SEM						
	2500		SEM						
	2500		SEM						
	2500		SEM						
	Not available		SEM						[1]
	10		SEM						[40]
	21	19.90% (RSD)	TEM						[41]
	32	30% (RSD)	TEM						
	46	22.20% (RSD)	TEM						
	299	15.60% (RSD)	TEM						
	76	10.80% (RSD)	TEM						
	190	19.20% (RSD)	TEM						
	448	16.70% (RSD)	TEM						
	658	25.40% (RSD)	TEM						
	331	20.50% (RSD)	TEM						
	449	16.10% (RSD)	TEM						
	563	17.40% (RSD)	TEM						
	1190	12.40% (RSD)	TEM						
	58.3		PXRD						[42]
	51.9		PXRD						
	41.7		PXRD						
	51.8		PXRD						
	70		PXRD						
	80.9		PXRD						
	60		PXRD						
	78.6		PXRD						

Table S3: Nanocrystal sizes used to create Fig 1 in the main text. Size distributions given in nanometers are shown in parentheses. Dispersity measurements reported as PDI (Polydispersity Index), standard deviation, relative standard deviation, or a range of nanometers are reported in Disp. (Other) with the type of data in parentheses (PDI, SD, RSD, or Range). In cases where anisotropic particles were presented with both length and width, they are differentiated here as (L) and (W).

MOF	Size (nm)	Disp. (Other)	Method	Size	Method	Size	Method	Method Average	Citation
	68.5		PXRD						
	42.4		PXRD						
	42.1		PXRD						
	57.4		PXRD						
	57		PXRD						
	Not available		TEM	2.2	PXRD	3	PXRD	2.6	[2]
	1.5		TEM	3.3	PXRD	4.7	PXRD	3.17	
	2		TEM	4.3	PXRD	6.1	PXRD	4.13	
	2.5		TEM	6.2	PXRD	8.7	PXRD	5.8	
IR-MOF-3	30		DLS						[6]
	32		DLS						
	33		DLS						
	47.5		DLS						
	46		DLS						
	Not available								[72]
Hf-BUT-12	Not available								[98]
Hf-UiO-66-NH_2	Not available								[98]
Hf-UiO-66-2,6-NDC	Not available								[98]
MFU-4	>1200		DLS						[7]
	>1200		DLS						
	>1200		DLS						
	>1200		DLS						
	>1200		DLS						
	>1200		DLS						
	>1200		DLS						
	119		DLS						
	46(2)		DLS	37(3)	PXRD	32(11)	TEM	38.33	
	42(1)		DLS	34(2)	PXRD	25(6)	TEM	33.67	
	48(1)		DLS	36(2)	PXRD	36(8)	TEM	40	
	43(3)		DLS	35(1)	PXRD	29(8)	TEM	35.67	
MIL-53	350(100)								[16]
MIL-53 Al	Not available								[65]
MIL-88B-Cr	Not available								[58]
MIL-88B-Fe	Not available								[60]
MIL-88B-Fe-NH_2	30		TEM						[12]
	55		TEM						
	155		TEM						
	160		TEM						
MIL-88A	100(25)		DLS						[16]
	Not available								[100]
	255(25)								[11]
	390(30)								
	360(35)								
	550(55)								
	110(25)		DLS						
	195(15)		DLS						
	230(25)		DLS						

Table S3: Nanocrystal sizes used to create Fig 1 in the main text. Size distributions given in nanometers are shown in parentheses. Dispersity measurements reported as PDI (Polydispersity Index), standard deviation, relative standard deviation, or a range of nanometers are reported in Disp. (Other) with the type of data in parentheses (PDI, SD, RSD, or Range). In cases where anisotropic particles were presented with both length and width, they are differentiated here as (L) and (W).

MOF	Size (nm)	Disp. (Other)	Method	Size	Method	Size	Method	Method Average	Citation
	150(30)		DLS						
	270(20)		DLS						
	290(20)		DLS						
	210(25)		DLS						
	380(35)		DLS						
	360(50)		DLS						
	275(65)		DLS						
	885(90)		DLS						
	1050(210)		DLS						
	720(75)		DLS						
	>1200		DLS						
	380(35)		DLS						
	480(45)		DLS						
	>1200		DLS						
	180(15)		DLS						
	240(15)		DLS						
	390(25)		DLS						
	95(15)		DLS						
	75(10)		DLS						
	190(20)		DLS						
	60(10)		DLS						
	70(10)		DLS						
	180(15)		DLS						
	115(15)		DLS						
	70(10)		DLS						
	75(10)		DLS						
	60(5)		DLS						
	80(5)		DLS						
	85(8)		DLS						
	110(10)		DLS						
	160(10)		DLS						
	270(23)		DLS						
	350(55)		DLS						
	87(15)		DLS						
	110(10)		DLS						
	280(45)		DLS						
	575(85)		DLS						
	110(10)		DLS						
	280(45)		DLS						
	575(85)		DLS						
	110(10)		DLS						
	185(25)		DLS						
	275(25)		DLS						
MIL-89	75(25)		DLS						[16]
MIL-96 Al	8500		Length	1000	Width				[13]
	2900		SEM (L)	450	SEM (W)				
	1600		SEM (L)	410	SEM (W)				

Table S3: Nanocrystal sizes used to create Fig 1 in the main text. Size distributions given in nanometers are shown in parentheses. Dispersity measurements reported as PDI (Polydispersity Index), standard deviation, relative standard deviation, or a range of nanometers are reported in Disp. (Other) with the type of data in parentheses (PDI, SD, RSD, or Range). In cases where anisotropic particles were presented with both length and width, they are differentiated here as (L) and (W).

MOF	Size (nm)	Disp. (Other)	Method	Size	Method	Size	Method	Method Average	Citation
	980		SEM (L)	510	SEM (W)				
	2100		SEM (L)	570	SEM (W)				
	2200		SEM (L)	810	SEM (W)				
	1290		SEM (L)	610	SEM (W)				
MIL-100 Al	585(150)		DLS (H_2O)	565(75)	DLS (EtOH)			575	[15]
	249(28)		DLS (H_2O)	237(41)	DLS (EtOH)	311(41)	DLS (DMEM)	272	[14]
	291(24)		DLS (MEM)						
MIL-100 Fe	100(50)		DLS						[16]
	452		DLS (Tris)	256	DLS (H_2O)			354	[52]
	646		DLS (Tris)					323	
	458		DLS (Tris)	215	DLS (H_2O)			336.5	
	698		DLS (Tris)					698	
	596		DLS (Tris)					596	
	592		DLS (Tris)	238	DLS (H_2O)			415	
	139(25)		DLS (H_2O)	168(10)	DLS (EtOH)			153.5	[15]
	110(25)		DLS (H_2O)	119(11)	DLS (EtOH)			114.5	
	141(43)		DLS (H_2O)	155(61)	DLS (PBS)	162(60)	DLS (PBS + Alb)	150.75	[101]
	145(38)		DLS (RMPI)						
	139(25)		DLS(H_2O)	168(10)	DLS (EtOH)	252(32)	DLS (DMEM)	203.5	[14]
	255(21)		DLS (MEM)						
MIL-100 Cr	143(63)		DLS (H2O)	80(41)	DLS (EtOH)	106(47)	DLS (MeOH)	109.33	[15]
	142(63)		DLS (H_2O)	80(41)	DLS (EtOH)	146(32)	DLS (DMEM)	130.25	[14]
	153(49)		DLS (MEM)						
MIL-101 Cr	50(9)		TEM						[17]
	19(4)		TEM						
	25(6)		TEM						
	28(6)		TEM						
	36(7)		TEM						
	73(8)		TEM						
	387(28)		TEM						[53]
	383(25)		TEM						
	346(19)		TEM						
	279(21)		TEM						
	160(16)		TEM						
	148(14)		TEM						
	90(10)		TEM						
	114(13)		SEM						[55]
	1336(174)		SEM						
	141(16)		SEM						
	87(9)		SEM						
	101(11)		SEM						
	214(25)		SEM						
	387(28)								[58]
	156(17)								
	100(12)								
MIL-101-Fe-NH_2	Not available								[40]
MIL-125	Not available								[62]

Table S3: Nanocrystal sizes used to create Fig 1 in the main text. Size distributions given in nanometers are shown in parentheses. Dispersity measurements reported as PDI (Polydispersity Index), standard deviation, relative standard deviation, or a range of nanometers are reported in Disp. (Other) with the type of data in parentheses (PDI, SD, RSD, or Range). In cases where anisotropic particles were presented with both length and width, they are differentiated here as (L) and (W).

MOF	Size (nm)	Disp. (Other)	Method	Size	Method	Size	Method	Method Average	Citation
	200(50)								[18]
MIL-125-NH_2	240(70)		DLS (DMF)					313.33	[19]
	660(250)		DLS (H_2O)						
	320(100)		DLS (MeOH)						
	220(60)		DLS (EtOH)						
	230(60)		DLS (PBS)						
	210(100)		DLS (PBS FBS)						
	750		SEM						[64]
	280		SEM						
	192		SEM						
	100		SEM						
	70		SEM						
	150		SEM						
	190		SEM						
	750		SEM						
	420		SEM						
	320		SEM						
	220		SEM						
	100		SEM						
	90		SEM						
	180		SEM						
	350		SEM						
	720		SEM						
MOF-5	Not available								[1]
	Not available								[40]
	70(10)		TEM						[70]
	624(39)								[71]
	399(23)								
	220	140-420							[72]
	125(25)								[73]
	37.5(7.5)								[74]
	150								
MOF-74	Not available								[40]
	17(3) (Co)								[9]
	100(20) (Mn)								
	200(50) (Mg)								
	18(5) (Ni)								
MOF-801	168(24)		SEM						[10]
	23(11)		SEM						
NU-1000	180	140-220 (Range)							[22]
	Not available								[77]
	75								[20]
	150								
	500								
	1200								
	15000								
	150(50)								[78]

Table S3: Nanocrystal sizes used to create Fig 1 in the main text. Size distributions given in nanometers are shown in parentheses. Dispersity measurements reported as PDI (Polydispersity Index), standard deviation, relative standard deviation, or a range of nanometers are reported in Disp. (Other) with the type of data in parentheses (PDI, SD, RSD, or Range). In cases where anisotropic particles were presented with both length and width, they are differentiated here as (L) and (W).

MOF	Size (nm)	Disp. (Other)	Method	Size	Method	Size	Method	Method Average	Citation
	300(50)								
	600(100)								
	1500(500)								
NU-1003	300		SEM						[21]
	1000		SEM						
	2000		SEM						
	7000		SEM						
	10000		SEM						
PCN-222	190	150-250 (Range)							[22]
	350(50)								[13]
	475(25)								
	625(125)								
	650(50)								
	925(75)								
	Not available								[98]
PCN-224	33(4)		TEM	65(3)	DLS			49	[23]
	91(5)		TEM	114(3)	DLS			86.5	
	91(8)		TEM	137(2)	DLS			114	
	144(7)		TEM	197(1)	DLS			170.5	
	189(11)		TEM	255(2)	DLS			222	
	100	50-120 (Range)							[80]
	190	150-150 (Range)							[22]
UiO-66	106.5(11.9)		SEM	547.1(25.1)	DLS			326.8	[81]
	118.2(18.1)		SEM	1327(57.6)	DLS			722.6	
	90.2(10.9)		SEM	472.7(182.3)	DLS			281.45	
	66.9(10.2)		SEM	288.1(31.4)	DLS			177.5	
	71.4(10.2)		SEM	167.7	DLS			119.55	
	82.5(12.2)		SEM	526.2(24.1)	DLS			304.35	
	87.9(15.3)		SEM	872.5(9.2)	DLS			480.2	
	66.4(12.3)		SEM	374.2(33.9)	DLS			220.3	
	23.7(5.0)		SEM	244.7(4.1)	DLS			134.2	
	23.1(3.5)		SEM	139.9(8.7)	DLS			81.5	
	91.9(21.0)		SEM	443.3(54.7)	DLS			267.6	
	53(9.7)		SEM	709.2(40.7)	DLS			381.1	
	29.2(6.8)		SEM	388.1(99)	DLS			208.65	
	26.5(5.9)		SEM	209.2(33)	DLS			117.85	
	20.0(4.0)		SEM	132.2(6.2)	DLS			76.2	
	26.5(5.5)		SEM	294.8	DLS			160.65	
	82.5(12.2)		SEM	526.2(24.1)	DLS			304.35	
	23.3(3.1)		SEM	575	DLS			299.15	
	17(2)		STEM	Not available	DLS			17	[83]
	34(22)		STEM	Not available	DLS			34	
	72(18)		STEM	158(19)	DLS			115	
	208(56)		STEM	173(40)	DLS			190.5	
	270(48)		STEM	319(67)	DLS			294.5	
	514(88)		STEM	677(204)	DLS			595.5	
	721(56)		STEM	826(135)	DLS			773.5	

Table S3: Nanocrystal sizes used to create Fig 1 in the main text. Size distributions given in nanometers are shown in parentheses. Dispersity measurements reported as PDI (Polydispersity Index), standard deviation, relative standard deviation, or a range of nanometers are reported in Disp. (Other) with the type of data in parentheses (PDI, SD, RSD, or Range). In cases where anisotropic particles were presented with both length and width, they are differentiated here as (L) and (W).

MOF	Size (nm)	Disp. (Other)	Method	Size	Method	Size	Method	Method Average	Citation
	17(4)		STEM	83(38)	DLS			50	
	31(5)		STEM	89(25)	DLS			60	
	44(12)		STEM	94(19)	DLS			69	
	147(50)		STEM	175(31)	DLS			161	
	439(83)		STEM	559(110)	DLS			499	
	813(223)		STEM	919(144)	DLS			866	
	1174(258)		STEM	1208(275)	DLS			1191	
	19(5)		STEM	132(72)	DLS			75.5	
	29(8)		STEM	74(18)	DLS			51.5	
	88(25)		STEM	95(24)	DLS			91.5	
	550(51)		STEM	784(70)	DLS			667	
	38(7)		STEM	100(29)	DLS			69	
	53(12)		STEM	140(30)	DLS			96.5	
	227(46)		STEM	277(80)	DLS			252	
	1949	154 (SD)	SEM						[84]
	1060	65 (SD)	SEM						
	783	32 (SD)	SEM						
	583	19 (SD)	SEM						
	522	24 (SD)	SEM						
	502	26 (SD)	SEM						
	220	122-615 (Range)	DLS						[85]
	255	105-396 (Range)	DLS						
	164	142-459 (Range)	DLS						
	164	105-396 (Range)	DLS						
	105	79-255 (Range)	DLS						
	164	122-342 (Range)	DLS						
	190	164-458 (Range)	DLS						
	85		XRD						
	83		XRD						
	81		XRD						
	78		XRD						
	74		XRD						
	71		XRD						
	46		XRD						
	23		XRD						
	14		XRD						
	265		SEM						[86]
	96		SEM						
	48		SEM						
	155		SEM						
	75		SEM						
	40		SEM						
	190		SEM						
	153		SEM						
	144		SEM						
	117		SEM						
	224		SEM						

Table S3: Nanocrystal sizes used to create Fig 1 in the main text. Size distributions given in nanometers are shown in parentheses. Dispersity measurements reported as PDI (Polydispersity Index), standard deviation, relative standard deviation, or a range of nanometers are reported in Disp. (Other) with the type of data in parentheses (PDI, SD, RSD, or Range). In cases where anisotropic particles were presented with both length and width, they are differentiated here as (L) and (W).

MOF	Size (nm)	Disp. (Other)	Method	Size	Method	Size	Method	Method Average	Citation
	150		SEM						
	167		SEM						
	177		SEM						
	150		SEM						
	100		SEM						
	97		SEM						
	102		SEM						
	265		SEM						
	91		SEM						
	78		SEM						
	61		SEM						
	41		SEM						
	230		SEM						[87]
	Not available								[98]
UiO-66-NH_2	115		PXRD						[24]
	90		PXRD						
	85		PXRD						
	82		PXRD						
	80		PXRD						
	54		PXRD						
	21		PXRD						
	16		PXRD						
	200		TEM						[98]
UiO-66-OH	Not available								[98]
UiO-66-2,6-NDC	Not available								[98]
UiO-67	641	365-995 (Range)	DLS						[24]
	553	356-859 (Range)	DLS						
	553	356-859 (Range)	DLS						
	413	308-641 (Range)	DLS						
	308	229-478 (Range)	DLS						
	478	356-742 (Range)	DLS						
	Not availble								[98]
ZIF-7	112		DLS						[91]
	30.7(5.9)		TEM						[25]
	40(10)		SEM						[102]
	70(10)		SEM						
ZIF-8	18		PXRD	17	SLS			17.5	[26]
	45		PXRD	39	SLS			42	
	10		PXRD	9	SLS			9.5	
	24		PXRD	20	SLS			22	
	55		PXRD	40	SLS			47.5	
	9		PXRD	9	SLS			9	
	16		PXRD	16	SLS			16	
	43		PXRD	42	SLS			42.5	
	10		PXRD	8	SLS			9	
	50(20)								[103]
	44(17)								

Table S3: Nanocrystal sizes used to create Fig 1 in the main text. Size distributions given in nanometers are shown in parentheses. Dispersity measurements reported as PDI (Polydispersity Index), standard deviation, relative standard deviation, or a range of nanometers are reported in Disp. (Other) with the type of data in parentheses (PDI, SD, RSD, or Range). In cases where anisotropic particles were presented with both length and width, they are differentiated here as (L) and (W).

MOF	Size (nm)	Disp. (Other)	Method	Size	Method	Size	Method	Method Average	Citation
	34	15-65 (Range)							
	60(25)								
	21(4)		TEM						[90]
	744		TEM						[89]
	601		TEM						
	490		TEM						
	271		TEM						
	93		TEM						
	43		TEM						
	27		TEM						
	40.6	0.09 (PDI)	SLS						[95]
	45.4		SLS						
	28		SLS						
	31.2		SLS						
	25		SLS						
	25		SLS						
ZIF-65-Zn	125		DLS						[91]
	125		DLS						
ZIF-71	55		DLS						[91]
	83		DLS						
	83		DLS						
	98		PXRD	100	SEM			99	[104]
	11		PXRD	15	SEM			13	
	94		PXRD	90	SEM			92	
	44		PXRD	30	SEM			37	
	24		PXRD	25	SEM			24.5	
Zn-BPD-H	105(15)		DLS	46	PXRD			75.5	[28]
	150(20)		DLS	47	PXRD			98.5	
	145(15)		DLS	50	PXRD			97.5	
	155(30)		DLS	51	PXRD			103	
	140(20)		DLS	60	PXRD			100	
	170(25)		DLS	50	PXRD			110	
	180(25)		DLS	52	PXRD			151	
Zn-BDP-NH_2	190(35)		DLS	Not available				95	[28]
	210(30)		DLS	58	PXRD			134	
	193(40)		DLS	71	PXRD			132	
	220(35)		DLS	67	PXRD			143.5	
	210(35)		DLS	65	PXRD			137.5	
	210(35)		DLS	71	PXRD			140.5	
	275(35)		DLS	61	PXRD	120(40) (L), 45(20) (W)	SEM	228	
Zn-BPD-NO_2	125(35)		DLS	45				85	[28]
	100(30)		DLS	43	PXRD			71.5	
	120(25)		DLS	42	PXRD			81	
	120(30)		DLS	39	PXRD			79.5	
	115(30)		DLS	Not available				57.5	
	100(30)		DLS	37	PXRD			68.5	
	120(35)		DLS	39	PXRD	65(30)	SEM	79.5	

Table S3: Nanocrystal sizes used to create Fig 1 in the main text. Size distributions given in nanometers are shown in parentheses. Dispersity measurements reported as PDI (Polydispersity Index), standard deviation, relative standard deviation, or a range of nanometers are reported in Disp. (Other) with the type of data in parentheses (PDI, SD, RSD, or Range). In cases where anisotropic particles were presented with both length and width, they are differentiated here as (L) and (W).

MOF	Size (nm)	Disp. (Other)	Method	Size	Method	Size	Method	Method Average	Citation
Zn-BPD-OH	145(35)		DLS	52	PXRD			98.5	[28]
	145(35)		DLS	50	PXRD			97.5	
	160(30)		DLS	45	PXRD			102.5	
	165(35)		DLS	45	PXRD			105	
	165(35)		DLS	40	PXRD			102.5	
	160(35)		DLS	34	PXRD			97	
	155(40)		DLS	50	PXRD	60(20)		132.5	

References

[1] H. Guo, Y. Zhu, S. Wang, S. Su, L. Zhou and H. Zhang, *Chem. Mater.*, 2012, **24**, 444.

[2] X. Xu, *Nanoscale*, 2016, **8**, 16725.

[3] Y. Qi, C.-T. He, J. Lin, S. Lin, J. Liu, J. Huang, W. Xue, G. Yu, H.-Y. Chao, Y. Tong and Z. Qiao, *Nano Research*, 2017, **10**, 3621.

[4] W. Zheng, X. Hao, L. Zhao and W. Sun, *Ind. Eng. Chem. Res.*, 2017, **56**, 5899.

[5] X. G. Wang, Q. Cheng, Y. Yu and X. Z. Zhang, *Angew. Chem. Int. Ed.*, 2018, **57**, 7836.

[6] D. Li, H. Wang, X. Zhang, H. Sun, X. Dai, Y. Yang, L. Ran, X. Li, X. Ma and D. Gao, *Cryst. Growth Des.*, 2014, **14**, 5856.

[7] H. Bunzen, M. Grzywa, M. Hambach, S. Spirkl and D. Volkmer, *Cryst. Growth Des.*, 2016, **16**, 3190.

[8] D. Manuel, A. Mayoral, D. Isabel and M. Sa, *Cryst. Growth Des.*, 2014, **14**, 2479.

[9] J. E. Bachman, Z. P. Smith, T. Li, T. Xu and J. R. Long, *Nat. Mater.*, 2016, **15**, 845.

[10] I. Abánades Lázaro, S. Haddad, J. M. Rodrigo-Muñoz, C. Orellana-Tavra, V. Del Pozo, D. Fairen-Jimenez and R. S. Forgan, *ACS Appl. Mater. Interfaces*, 2018, **10**, 5255.

[11] T. Chalati, P. Horcajada, R. Gref, P. Couvreur and C. Serre, *J. Mater. Chem.*, 2011, **21**, 2220.

[12] M. Pham, G. Vuong, A. Vu and T. Do, *Langmuir*, 2011, **27**, 15261.

[13] D. Liu, Y. Liu, F. Dai, J. Zhao, K. Yang and C. Liu, *Dalton Trans.*, 2015, **96**, 16421.

[14] R. Grall, T. Hidalgo, J. Delic, A. Garcia-Marquez, S. Chevillard and P. Horcajada, *J. Mater. Chem. B*, 2015, **3**, 8279.

[15] A. G. Márquez, A. Demessence, A. E. Platero-prats, D. Heurtaux, P. Horcajada, C. Serre, J. Chang, G. Férey, A. De, C. Boissière, D. Grosso and C. Sanchez, *Eur. J. Inorg. Chem.*, 2012, **100**, 5165.

[16] T. Chalati, P. Horcajada, P. Couvreur, C. Serre, M. Ben Yahia, G. Maurin and R. Gref, *Nanomedicine*, 2011, **6**, 1683.

[17] D. Jiang, A. D. Burrows and K. J. Edler, *CrystEngComm*, 2011, **13**, 6916.

[18] C. T. Saouma, S. Richard, S. Smolders, M. F. Delley, R. Ameloot, F. Vermoortele, D. E. De Vos and J. M. Mayer, *J. Am. Chem. Soc.*, 2018, **140**, 16184.

[19] S. Vilela, P. Salcedo-Abraira, I. Colinet, F. Salles, M. de Koning, M. Joosen, C. Serre and P. Horcajada, *Nanomaterials*, 2017, **7**, 321.

[20] P. Li, R. C. Klet, S.-y. Moon, T. C. Wang, P. Deria, A. W. Peters, B. M. Klahr, H.-j. Park, S. S. Al-juaid, J. T. Hupp and O. K. Farha, *Chem. Comm.*, 2015, **51**, 10925.

[21] S. P. Harvey, J. T. Hupp and O. K. Farha, *ACS Nano*, 2016, **10**, 9174.

[22] S. Wang, Y. Chen, S. Wang, P. Li, C. A. Mirkin and O. K. Farha, *J. Am. Chem. Soc.*, 2019, **141**, 2215.

[23] J. Park, Q. Jiang, D. Feng, L. Mao and H. Zhou, *J. Am. Chem. Soc.*, 2016, **138**, 3518.

[24] A. Schaate, P. Roy, A. Godt, J. Lippke, F. Waltz, M. Wiebcke and P. Behrens, *Chem. Eur. J.*, 2011, **17**, 6643.

[25] Y. S. Li, H. Bux, A. Feldhoff, G. N. Li, W. S. Yang and J. Caro, *Adv. Mater.*, 2010, **22**, 3322.

[26] J. Cravillon, R. Nayuk, S. Springer, A. Feldhoff, K. Huber and M. Wiebcke, *Chem. Mater.*, 2011, **23**, 2130.

[27] I. H. Lim, W. Schrader and F. Schüth, *Chem. Mater.*, 2015, **27**, 3088.

[28] S. Rojas, F. J. Carmona, C. R. Maldonado, P. Horcajada, T. Hidalgo, C. Serre, J. A. Navarro and E. Barea, *Inorg. Chem.*, 2016, **55**, 2650.

[29] A. Chouhan, G. Pilet, S. Daniele and A. Pandey, *Chinese J. Chem.*, 2017, **35**, 209.

[30] J. Enríquez, C. Manquian, I. Chi-Duran, F. Herrera and D. P. Singh, *ACS Omega*, 2019, **4**, 7411.

[31] Y.-y. Liu, R. Decadt, T. Bogaerts, K. Hemelsoet, A. M. Kaczmarek, D. Poelman, M. Waroquier, V. V. Speybroeck, R. V. Deun and P. V. D. Voort, *J. Phys. Chem. C*, 2013, **117**, 11302.

[32] Z. Qi, J. Yang, Y. Kang, F. Guo and W. Sun, *Dalton Trans.*, 2016, **45**, 8753.

[33] N. Klein, I. Senkovska, I. A. Baburin, R. Grünker, U. Stoeck, M. Schlichtenmayer, B. Streppel, U. Mueller, S. Leoni, M. Hirscher and S. Kaskel, *Chem. Eur. J.*, 2011, **17**, 13007.

[34] Q. Zhou, F. Yang, B. Xin, G. Zeng, X. Zhou, K. Liu, D. Ma, G. Li, Z. Shi and S. Feng, *Chem. Commun.*, 2013, **49**, 8244.

[35] X. Cai, B. Liu, M. Pang and J. Lin, *Dalton Trans.*, 2018, **47**, 16329.

[36] X. Cai, Z. Xie, M. Pang and J. Lin, *Cryst. Growth Des.*, 2019, **19**, 556.

[37] S. Xiang, W. Zhou, J. M. Gallegos, Y. Liu and B. Chen, *J. Am. Chem. Soc.*, 2009, **131**, 12415.

[38] F. Wang, H. Guo, Y. Chai, Y. Li and C. Liu, *Microporous Mesoporous Mater.*, 2013, **173**, 181.

[39] J. L. Zhuang, D. Ceglarek, S. Pethuraj and A. Terfort, *Adv. Funct. Mater.*, 2011, **21**, 1442.

[40] C. Guo, Y. Zhang, Y. Guo, Y. Zhang and W. Jide, *Chem. Commun.*, 2018, **54**, 252.

[41] S. Diring, S. Furukawa, Y. Takashima, T. Tsuruoka and S. Kitagawa, *Chem. Mater.*, 2010, **22**, 4531.

[42] C. Xin, H. Zhan, X. Huang, H. Li, N. Zhao, F. Xiao, W. Wei and Y. Sun, *RSC Advances*, 2015, **5**, 27901.

[43] A. Ranft, S. B. Betzler, F. Haase and B. V. Lotsch, *CrystEngComm*, 2013, **15**, 9296.

[44] R. M. Abdelhameed, L. D. Carlos, A. M. Silva and J. Rocha, *Chem. Commun.*, 2013, **49**, 5019.

[45] S. Biswas, M. Grzywa, H. P. Nayek, S. Dehnen, I. Senkovska, S. Kaskel and D. Volkmer, *Dalton Trans.*, 2009, **9226**, 6487.

[46] M. Graf, N. Moreno, K. Kerl and H. Bunzen, *J. Mater. Chem.*, 2018, **6**, 6481.

[47] D. Denysenko, M. Grzywa, M. Tonigold, B. Streppel, I. Krkljus, M. Hirscher, E. Mugnaioli, U. Kolb, J. Hanss and D. Volkmer, *Chem. Eur. J.*, 2011, **17**, 1837.

[48] C. Volkringer, D. Popov, T. Loiseau, G. Férey, M. Burghammer, C. Riekel, M. Haouas and F. Taulelle, *Chem. Mater.*, 2009, **21**, 5695.

[49] M. Qiu, C. Chen and W. Li, *Catalysis Today*, 2015, **258**, 132.

[50] G. Férey, C. Serre, C. Mellot-Draznieks, F. Millange, S. Surblé, J. Dutour and I. Margiolaki, *Angew. Chem. Int. Ed.*, 2004, **43**, 6296.

[51] Y. K. Seo, J. W. Yoon, J. S. Lee, U. H. Lee, Y. K. Hwang, C. H. Jun, P. Horcajada, C. Serre and J. S. Chang, *Microporous Mesoporous Mater.*, 2012, **157**, 137.

[52] R. Anand, F. Borghi, F. Manoli, I. Manet, V. Agostoni, P. Reschiglian, R. Gref and S. Monti, *J. Phys. Chem. B*, 2014, **118**, 8532.

[53] T. Zhao, L. Yang, P. Feng, I. Gruber, C. Janiak and Y. Liu, *Inorganica Chim. Acta*, 2018, **471**, 440.

[54] M. Suresh, B. DAVID Raju, K. S. Rama Rao, K. Raveendranath Reddy, M. L. Kantam and P. Srinvasu, *J. of Chem. Sci.*, 2014, **126**, 527.

[55] T. Zhao, S. Li, L. Shen, Y. Wang and X. Yang, *Inorg. Chem. Commun.*, 2018, **96**, 47.

[56] L. Yang, T. Zhao, I. Boldog, C. Janiak, X. Yang, Q. Li, Y. Zhou, Y. Xia, D. Lai and Y. Liu, *Dalton Trans.*, 2019, **48**, 989.

[57] J. Wang, J. Wan, Y. Ma, Y. Wang, M. Pu and Z. Guan, *RSC Advances*, 2016, **6**, 112502.

[58] L. Yang, T. Zhao, I. Boldog, C. Janiak, X.-Y. Yang, Q. Li, Y.-J. Zhou, Y. Xia, D.-W. Lai and Y.-J. Liu, *Dalton Trans.*, 2019, **48**, 989.

[59] Y. H. Shih, S. H. Lo, N. S. Yang, B. Singco, Y. J. Cheng, C. Y. Wu, I. H. Chang, H. Y. Huang and C. H. Lin, *ChemPlusChem*, 2012, **77**, 982.

[60] X. Cai, J. Lin and M. Pang, *Cryst. Growth Des.*, 2016, **16**, 3565.

[61] M. Ma, A. Bétard, I. Weber, N. S. Al-Hokbany, R. A. Fischer and N. Metzler-Nolte, *Cryst. Growth. Des.*, 2013, **13**, 2286.

[62] W. S. Chi, D. K. Roh, C. S. Lee and J. H. Kim, *J. Mater. Chem. A*, 2015, **3**, 21599.

[63] S. Kim, J. Kim, H. Kim, H. Cho and W. Ahn, 2013, **204**, 85.

[64] S. Hu, M. Liu, X. Guo, K. Li, Y. Han, C. Song and G. Zhang, *Cryst. Growth. Des.*, 2017, **17**, 6586.

[65] Y. Liu and X. Zhao, *CrystEngComm*, 2018, **20**, 2102.

[66] Y. Xiao, T. Han, G. Xiao, Y. Ying, H. Huang, Q. Yang, D. Liu and C. Zhong, *Langmuir*, 2014, **30**, 12229.

[67] S. Han and M. S. Lah, *Cryst. Growth. Des.*, 2015, **15**, 5568.

[68] J. S. Choi, W. J. Son, J. Kim and W. S. Ahn, *Microporous Mesoporous Mater.*, 2008, **116**, 727.

[69] E. Burgaz, A. Erciyes, M. Andac and O. Andac, *Inorganica Chim. Acta*, 2019, **485**, 118.

[70] R. Nayuk, D. Zacher, R. Schweins, C. Wiktor, R. A. Fischer, G. Van Tendeloo and K. Huber, *Journal of Physical Chemistry C*, 2012, **116**, 6127.

[71] S. Wang, Y. Lv, Y. Yao, H. Yu and G. Lu, *Inorg. Chem. Commun.*, 2018, **93**, 56.

[72] M. Ma, D. Zacher, X. Zhang, R. A. Fischer and N. Metzler-Nolte, *Cryst. Growth Des.*, 2011, **11**, 185.

[73] L. Huang, H. Wang, J. Chen, Z. Wang, J. Sun, D. Zhao and Y. Yan, *Microporous Mesoporous Mater.*, 2003, **58**, 105.

[74] S. Hermes, T. Witte, T. Hikov, D. Zacher, S. Bahnmüller, G. Langstein, K. Huber and R. A. Fischer, *J. Am. Chem. Soc.*, 2007, **129**, 5324.

[75] F. S. Nesse, M. Opitz and P. D. C. Dietzel, *Microporous Mesoporous Mater.*, 2019, **275**, 207.

[76] T. Islamoglu, K. I. Otake, P. Li, C. T. Buru, A. W. Peters, I. Akpinar, S. J. Garibay and O. K. Farha, *CrystEngComm*, 2018, **20**, 5913.

[77] T. E. Webber, W. Liu, S. P. Desai, C. C. Lu, D. G. Truhlar and R. L. Penn, *ACS Appl. Mater. Interfaces*, 2017, **9**, 39342.

[78] A. Pankajakshan, M. Sinha, A. A. Ojha and S. Mandal, *ACS Omega*, 2018, **3**, 7832.

[79] G. Y. Zhang, Y. H. Zhuang, D. Shan, G. F. Su, S. Cosnier and X. J. Zhang, *Anal. Chem.*, 2016, **88**, 11207.

[80] J. Wang, Y. Fan, Y. Tan, X. Zhao, Y. Zhang, C. Cheng and M. Yang, *ACS Appl. Mater Interfaces*, 2018, **10**, 36615.

[81] G. E. Decker, Z. Stillman, L. Attia, C. A. Fromen and E. D. Bloch, *Chem. Mater.*, 2019, **31**, 4831.

[82] J. H. Cavka, S. Jakobsen, U. Olsbye, N. Guillou, C. Lamberti, S. Bordiga and K. P. Lillerud, *J. Am. Chem. Soc.*, 2008, **130**, 13850.

[83] W. Morris, S. Wang, D. Cho, E. Auyeung, P. Li, O. K. Farha and C. A. Mirkin, *ACS Appl. Mater. Interfaces*, 2017, **9**, 33413.

[84] Y. Zhao, Q. Zhang, Y. Li, R. Zhang and G. Lu, *ACS Appl. Mater. Interfaces*, 2017, **9**, 15079.

[85] L. A. Lozano and B. M. C. Faroldi, *J. Mater. Sci.*, 2018, 1862.

[86] Y. Han, M. Liu, K. Li, Q. Sun, C. Song, G. Zhang, Z. Zhang and X. Guo, *Cryst. Growth Des.*, 2017, **17**, 685.

[87] J. Cao, Z. Yang, W. Xiong, Y. Zhou, Y. Peng and X. Li, *Chem. Eng. J.*, 2018, **353**, 126.

[88] T. H. Park, K. A. Cychosz, A. G. Wong-Foy, A. Dailly and A. J. Matzger, *Chem. Commun.*, 2011, **47**, 1452.

[89] X. Lan, N. Huang, J. Wang and T. Wang, *Chem. Commun.*, 2018, **54**, 584.

[90] J. K. Zarba, M. Nyk and M. Samoć, *Cryst. Growth Des.*, 2016, **16**, 6419.

[91] M. Tu, C. Wiktor, C. Ro and R. A. Fischer, *Chem. Commun.*, 2014, **50**, 13258.

[92] A. Orsi, D. J. Price, J. Kahr, R. S. Pillai, S. Sneddon, S. Cao, V. Benoit, M. M. Łozińska, D. B. Cordes, A. M. Slawin, P. L. Llewellyn, I. Casely, S. E. Ashbrook, G. Maurin and P. A. Wright, *CrystEngComm*, 2017, **19**, 1377.

[93] P. Zhao, G. I. Lampronti, G. O. Lloyd, M. T. Wharmby, S. Facq, A. K. Cheetham and S. A. Redfern, *Chem. Mater.*, 2014, **26**, 1767.

[94] R. P. Lively, M. E. Dose, J. A. Thompson, B. A. McCool, R. R. Chance and W. J. Koros, *Chem. Commun.*, 2011, **47**, 8667.

[95] J. Cravillon, S. Münzer, S. Lohmeier, A. Feldhoff and K. Huber, *Chem. Mater.*, 2009, **21**, 1.

[96] K. S. Park, Z. Ni, A. P. Cote, J. Y. Choi, R. Huang, F. J. Uribe-Romo, H. K. Chae, M. O'Keeffe and O. M. Yaghi, *P. Natl. Acad. Sci.*, 2006, **103**, 10186.

[97] V. Colombo, C. Montoro, A. Maspero, G. Palmisano, N. Masciocchi, S. Galli, E. Barea and J. A. Navarro, *J. Am. Chem. Soc.*, 2012, **134**, 12830.

[98] T. He, X. Xu, B. Ni, H. Wang, Y. Long, W. Hu and X. Wang, *Nanoscale*, 2017, 19209.

[99] F. Wang, H. Guo, Y. Chai, Y. Li and C. Liu, *Microporous Mesoporous Mater.*, 2013, **173**, 181.

[100] E. Bagherzadeh, S. M. Zebarjad and H. R. M. Hosseini, *Eur. J. Inorg. Chem.*, 2018, **2018**, 1909.

[101] E. Bellido, T. Hidalgo, M. V. Lozano, M. Guillevic, R. Simón-Vázquez, M. J. Santander-Ortega, Á. González-Fernández, C. Serre, M. J. Alonso and P. Horcajada, *Adv. Healthc. Mater.*, 2015, **4**, 1246.

[102] L. Xiang, L. Sheng, C. Wang, L. Zhang, Y. Pan and Y. Li, *Adv. Mater.*, 2017, **29**, 1.

[103] T. Xing, Y. Lou, Q. Bao and J. Chen, *CrystEngComm*, 2014, **16**, 8994.

[104] S. Springer, A. Satalov, J. Lippke and M. Wiebcke, *Microporous Mesoporous Mater.*, 2015, **216**, 161.
